# Inhibiting DPP4 in a mouse model of HHT1 results in a shift towards regenerative macrophages and reduces fibrosis after myocardial infarction

**DOI:** 10.1371/journal.pone.0189805

**Published:** 2017-12-18

**Authors:** Calinda K. E. Dingenouts, Wineke Bakker, Kirsten Lodder, Karien C. Wiesmeijer, Asja T. Moerkamp, Janita A. Maring, Helen M. Arthur, Anke M. Smits, Marie-José Goumans

**Affiliations:** 1 Department of Molecular Cell Biology, Leiden University Medical Center, Leiden, the Netherlands; 2 Institute of Genetic Medicine, Newcastle University, International Centre for Life, Newcastle upon Tyne, United Kingdom; University of Kansas Medical Center, UNITED STATES

## Abstract

**Aims:**

Hereditary Hemorrhagic Telangiectasia type-1 (HHT1) is a genetic vascular disorder caused by haploinsufficiency of the TGFβ co-receptor endoglin. Dysfunctional homing of HHT1 mononuclear cells (MNCs) towards the infarcted myocardium hampers cardiac recovery. HHT1-MNCs have elevated expression of dipeptidyl peptidase-4 (DPP4/CD26), which inhibits recruitment of CXCR4-expressing MNCs by inactivation of stromal cell-derived factor 1 (SDF1). We hypothesize that inhibiting DPP4 will restore homing of HHT1-MNCs to the infarcted heart and improve cardiac recovery.

**Methods and results:**

After inducing myocardial infarction (MI), wild type (WT) and endoglin heterozygous (*Eng*^+/-^) mice were treated for 5 days with the DPP4 inhibitor Diprotin A (DipA). DipA increased the number of CXCR4^+^ MNCs residing in the infarcted *Eng*^+/-^ hearts (*Eng*^+/-^ 73.17±12.67 vs. *Eng*^+/-^ treated 157.00±11.61, P = 0.0003) and significantly reduced infarct size (*Eng*^+/-^ 46.60±9.33% vs. *Eng*^+/-^ treated 27.02±3.04%, P = 0.03). Echocardiography demonstrated that DipA treatment slightly deteriorated heart function in *Eng*^+/-^ mice. An increased number of capillaries (*Eng*^+/-^ 61.63±1.43 vs. *Eng*^+/-^ treated 74.30±1.74, P = 0.001) were detected in the infarct border zone whereas the number of arteries was reduced (*Eng*^+/-^ 11.88±0.63 vs. *Eng*^+/-^ treated 6.38±0.97, P = 0.003). Interestingly, while less M2 regenerative macrophages were present in *Eng*^+/-^ hearts prior to DipA treatment, (WT 29.88±1.52% vs. *Eng*^+/-^ 12.34±1.64%, P<0.0001), DPP4 inhibition restored the number of M2 macrophages to wild type levels.

**Conclusions:**

In this study, we demonstrate that systemic DPP4 inhibition restores the impaired MNC homing in *Eng*^+/-^ animals post-MI, and enhances cardiac repair, which might be explained by restoring the balance between the inflammatory and regenerative macrophages present in the heart.

## Introduction

Hereditary Hemorrhagic Telangiectasia type 1 (HHT1) is a haploinsufficient genetic vascular disorder caused by mutations in the transforming growth factor beta (TGFβ) co-receptor endoglin (CD105). HHT1 is characterized by 'leaky' vessel formation due to impaired TGFβ signaling, demonstrated by internal organ bleeding, endothelial hyperplasia, arteriovenous malformations (AVMs) and recurrent epistaxis, up to 8 times a day [1, 2]. Interestingly, endoglin heterozygous mice (*Eng*^+/-^) can develop the same clinical features as HHT1 patients [3, 4]. In time, depending on the genetic background [5], *Eng*^+/-^ mice develop AVMs clearly visible in the ear, and even suffer from nosebleeds, making the *Eng*^+/-^ mouse a good experimental model to gain more insight in the etiology of HHT1.

The partial loss of endoglin results in reduced angiogenesis [6], and defective collateral artery formation after hind limb ischemia in *Eng*^+/-^ mice [7], emphasizing the necessity of sufficient endoglin expression for proper revascularization after tissue damage [8, 9]. Although endoglin is mainly expressed by endothelial cells (ECs), several other cell types including smooth muscle cells (SMCs) and mononuclear cells (MNCs) have endoglin on their cell surface [10]. Due to the prominent role of endoglin in endothelial cell signaling and behavior, HHT1 is generally considered a result of endothelial dysfunction. However, it has become clear that the other cell types expressing endoglin, e.g. immune cells, may have a severe impact on angiogenesis and tissue repair as well [11–13].

Previously we showed that *Eng*^+/-^ mice display a diminished cardiac recovery after experimentally induced myocardial infarction (MI) compared to wild type littermates [14]. The reduced cardiac function is partially rescued when human control MNCs were injected into the tail vein of *Eng*^+/-^ mice. Interestingly, delivery of MNCs isolated from peripheral blood of HHT1 patients did not increase heart function post-MI. Furthermore, endoglin heterozygosity reduced the homing capacity of MNCs to the injured myocardium due to enhanced dipeptidyl peptidase-4 (DPP4, also known as CD26) expression levels [14, 15]. This suggests that a defect in MNCs, and not only endothelial cells, may play a role in the pathology of HHT1.

During the cardiac post-injury response, homing of MNCs to the site of injury is regulated by the stromal cell-derived factor 1 (SDF1)-CXC chemokine receptor type 4 (CXCR4) axis. SDF1 levels are increased within the first 24 hours after MI [16]. MNCs expressing the SDF1 receptor CXCR4 respond to this gradient by homing to the site of injury. The SDF1-CXCR4 axis is tightly controlled by the negative regulator DPP4. The catalytic enzyme DPP4 inactivates SDF1 by cleaving off the first two of its amino-terminal peptides, thereby decreasing the ability to recruit CXCR4-expressing MNCs towards the SDF1 gradient [17].

Another important target of DPP4 is the glucagon-like peptide-1 (GLP1). Preserving GLP1 via DPP4 inhibition stimulates insulin secretion and has therefore been the recent focus for treatment of type 2 diabetes mellitus (T2DM). Interestingly, DPP4 inhibition was shown to have multiple off-target effects that are potentially beneficial in the treatment of cardiovascular disease [18, 19]. These DPP4 inhibitory actions are GLP1 independent and can range from having an anti-inflammatory effect [20, 21] to having a stimulating effect on MNC migration [15] and differentiation [22, 23]. Pre-treating MNCs of HHT1 patients with the DPP4 inhibitor Diprotin A (DipA) before injection into the circulation restored their homing to the ischemic myocardium [15]. Unfortunately, this is not a suitable protocol for clinical practice to pre-treat MNCs of HHT1 patients, as cells have to be isolated in sufficient numbers from the patients’ peripheral blood, treated with a DPP4 inhibitor and re-injected. Therefore, the aim of this study is to determine whether systemic application of a DPP4 inhibitor is as effective in restoring homing of *Eng*^*+/-*^ MNCs to the site of ischemic injury.

## Materials & methods

### Animals and study design

Experiments and analyses were conducted on male endoglin wild type (*Eng*^*+/+*^, or referred to as WT) and heterozygous (*Eng*^*+/-*^) transgenic mice and LysM-Cre-*Eng*^fl/+^ / LysM-Cre-*Eng*^fl/fl^ (endoglin targeted recombination under regulation of the Lysosome M promoter) transgenic mice. All mouse strains were kept on a C57BL/6Jico background (Charles River). To obtain the *Eng*-conditional knockout mouse lines, endoglin floxed mice (*Eng*^fl/fl^) were cross-bred with LysM-Cre [24, 25] animals to create the LysM-Cre-*Eng*^fl/+^ and LysM-Cre-*Eng*^fl/fl^ mice. All mouse experiments were approved by the regulatory authorities of Leiden University (the Netherlands) and were in compliance with the guidelines from Directive 2010/63/EU of the European Parliament on the protection of animals used for scientific purposes.

Humane endpoints were observed 5 days post-MI and onwards, as the following criteria and symptoms: when mice displayed reduced mobility, decreased grooming, and/or impaired reaction to external stimuli. In addition, for 3 days post-MI and onwards: when the wound area displayed bleeding, swelling, redness and/or discharge, the mice would be euthanized by carbon dioxide. The mice were weighed at the day of surgery and at the cardiac ultrasound time points and euthanized when more than 15% loss of weight occurred. All mice that died before meeting the criteria for euthanasia–just after myocardial infarction or within 10 days post-MI–died because of cardiac rupture due to the deterioration of cardiac tissue after ligation of the left anterior descending coronary artery. Animal health and behavior were monitored on a daily basis by the research and/or animal care staff, all trained in animal care and handling. Once animals reached endpoint criteria, the euthanasia was performed immediately or at the least the same day when reported.

### Myocardial infarction in mice

Myocardial infarction (MI) was experimentally induced as described before [15]. The mice (n = 5–18 per group) were anesthetized with isoflurane (1.5–2.5%), intubated and ventilated, after which the left anterior descending (LAD) coronary artery was permanently ligated by placement of a suture. The mice were treated with the analgesic drug Temgesic, both pre-operative and 24 hrs post-operative to relieve pain. The mice were randomly allocated and treated i.p. with either 100 μl distilled water daily (Milli-Q ultrapure, sterile water = MQ treated or control group) or 100 μl DPP4 inhibitor (5 nMol, 55 μg/kg/day, Diprotin A, Sigma-Aldrich) for the first 5 or 14 days post-MI.

### Cardiac function measurements

Mice were anesthetized with isoflurane (1.5–2.5%), after which cardiac ultrasound was performed and recorded with the Vevo 770 (VisualSonics, Inc., Toronto, CA) system, using a 30 MHz transducer (RMV707B). Imaging was performed on the longitudinal axis of the left ventricle using the EKV (Electrocardiography-based Kilohertz Visualization) in long axis view imaging mode. The percentage ejection fraction was determined by tracing of the volume of the left ventricle during the systolic and diastolic phase using the imaging software Vevo770 V3.0 (VisualSonics, Inc., Toronto, CA).

### Cultured macrophages from mouse bone marrow

Monocytes were isolated from the mice femur and tibia and subsequently cultured in RPMI 1640 culture media (#11875093, Gibco, ThermoFisher Scientific), supplemented with 10% FBS (#10270, Fetal Bovine Serum, Gibco, ThermoFisher Scientific) and 1 ng/ml granulocyte-macrophage colony stimulating factor (GM-CSF, #315–03, Peprotech) to induce differentiation into macrophages. Macrophage cultures from 3 mice of each genotype were pooled to obtain sufficient protein to perform Western blot analysis.

### Western blotting

Cultured macrophages were lysed on ice with cold radio immunoprecipitation assay (RIPA) lysis buffer (in house) supplemented with protease inhibitors (Complete protease inhibitor cocktail tablets, Roche Diagnostics, #11697498001) and protein concentration was measured using BCA protein assay (Pierce BCA Protein Assay Kit, #23225, ThermoFisher Scientific). Equal amounts of protein were loaded onto 10% SDS-polyacrylamide gel and transferred to an Immobilon-P transfer membrane (# IPVH00010, PVDF membrane, Millipore). The blots were blocked for 1 h using 10% milk in Tris-Buffered Saline and 0.1% Tween-20 solution and incubated O/N with goat anti-mouse endoglin (1:1000 dilution, BAF1097, R&D Systems) or mouse anti-β-Actin (1:10.000 dilution, A5441, Sigma-Aldrich). Blots were incubated for 1 h with horse radish peroxidase anti-goat (goat anti-mouse IgG Poly-HRP Secondary Antibody HRP conjugate, #32230, ThermoFisher Scientific) or anti-mouse (ECL mouse IgG, HRP-linked whole Ab #NA931, Sigma-Aldrich, GE Healthcare, UK). Blots were developed in a Kodak X-omat 1000 processor with Thermo Scientific SuperSignal West Dura (Extended Duration Substrate) or SuperSignal West Pico and exposed to Fuji SuperRX medical X-ray film. Analysis was performed using Image J (National Institute of Mental Health, Bethesda, Maryland, USA).

### Immunofluorescence and immunohistochemistry

Hearts were dissected from carbon dioxide-euthanized mice, 4, 14 or 28 days post-MI, fixated overnight at 4°C in 4% paraformaldehyde in PBS, and then washed with PBS, 50% EtOH and 70% EtOH for 1 h each, followed by embedding in paraffin wax. Sections of 6 μm thickness were mounted onto coated glass slides (VWR SuperFrost Plus microscope slides). The paraffin sections were stained as previously described [26] using antigen retrieval. Primary antibodies were incubated overnight at 4°C and directed against rat anti-mouse CXCR4 (clone 2B11, dilution 1:100, # 551852, BD Pharmingen), rat anti-mouse MAC3 (CD107b, dilution 1:200, #550292, BD Biosciences), rabbit anti-mouse Mannose Receptor (CD206, dilution 1:300, ab64693, Abcam), rabbit anti-mouse alpha smooth muscle actin (αSMA, dilution 1:500, ab5694, Abcam), rat anti-mouse PECAM-1 (CD31, dilution 1:800, #TLD-4E8, BD Pharmingen) and goat anti-mouse cardiac troponin I (cTnI, 1:1000 dilution, #4T21, HyTest). Appropiate fluorescent-labelled secondary antibodies (ThermoFisher Scientific) were incubated for 1.5 h, at 1:250 dilutions. The slides were mounted with Prolong Gold-DAPI Antifade (# P36931, ThermoFisher Scientific) reagent.

Staining of fibrotic tissue was performed using Picrosirius Red (PSR) collagen staining which includes deparaffinization, 1 h incubation with PSR solution (Sirius red F3B, CI 35780, Sigma Aldrich, in Picrine acid solution, # 690550, Klinipath), washing in acidified water and mounting with Entellan (#107960, Merck) reagent. Staining of macrophages present in the infarct border zone using rat anti-mouse MAC3 (CD107b, dilution 1:200, BD Biosciences) was performed using avidin/biotin-based DAB peroxidase staining with the Vectastain ABC system (Vector Laboratories) and hematoxylin counterstain to visualize cell nuclei.

### Flow cytometry

After isolation of the hearts (n = 3–6), the left ventricle was excised and washed with PBS. Heart tissue was digested in collagenase I (450 U/ml), collagenase XI (125 U/ml), DNase I (60 U/ml) and hyaluronidase (60 U/ml) (Sigma-Aldrich #H3506) at 37°C for 1 h. Hearts were subsequently homogenized through a 100-μm cell strainer (VWR-Corning #10054–458). MNCs were isolated using Ficoll gradient (Histopaque-1083, Sigma, # 10831). Mouse MNCs from either 50 μL of whole blood (treated 5min with erythrocyte lysis buffer, #930725, Alrijne hospital Leiden), or cells isolated from heart, bone marrow and spleen were labeled 1 h at room temperature with anti-mouse CD11b (1:1600, BD Biosciences, #561114) and Ly6C (1:800, BD Biosciences, #561085) for macrophages. Macrophages were identified as inflammatory M1: Ly6G^-^/CD11b^+^/Ly6C^high^ and regenerative M2 macrophages: Ly6G^-^/CD11b^+^/Ly6C^low^ as previously described [27]. Lymphocytes subsets were labeled with CD3e (1:800, BD Biosciences, #558214), CD4 (1:800, Invitrogen, #MCD0422), CD8a (1:1600, BD Biosciences, #553032) and Ly6G (1:1600, BD Biosciences, #560602) in buffer (2mM EDTA/ 0.5% BSA in PBS).

All acquisitions were performed on a LSRII flow cytometer (BD Biosciences) and analyzed by FACS Diva software (BD Biosciences) and Flowing software 2.5.1 (Cell Imaging Core, Turku Centre for Biotechnology, Finland). Gating strategies are provided in S7 Fig.

### Morphometry

Infarct size was determined in Picrosirius Red staining images by calculating the percentage infarct area of the total left ventricular area. Cell infiltration was determined by quantification of 2 to 4 digital images per heart, at the border zone inside the infarcted area, taken at 40x magnification (CaseViewer 3D Histech). The same method was used for quantification of capillary and artery presence, except analysis was now at the outside borderzone of the infarct, where cardiomyocytes were still viable. Data were blinded to the investigator and quantified by using ImageJ v1.46r (NIH, USA).

### Statistics

All results are expressed as mean ± standard error of the mean (SEM). Statistical significance was accepted at p<0.05. Statistical significance was evaluated using one-way ANOVA testing for difference between multiple groups. Adjustment for multiple comparisons with either Tukey’s or Dunnett’s testing and unpaired students T-testing for testing between two groups, using Graphpad Prism v6 for Windows. For data with groups where n = 3, non-parametric testing was performed with Kruskal-Wallis ANOVA and Dunn’s multiple comparisons test. Significant differences between survival curves were tested with a log-rank Mantel-Cox test.

## Results

### Involvement of macrophage-expressed endoglin during cardiac regeneration

**Fig 1:**
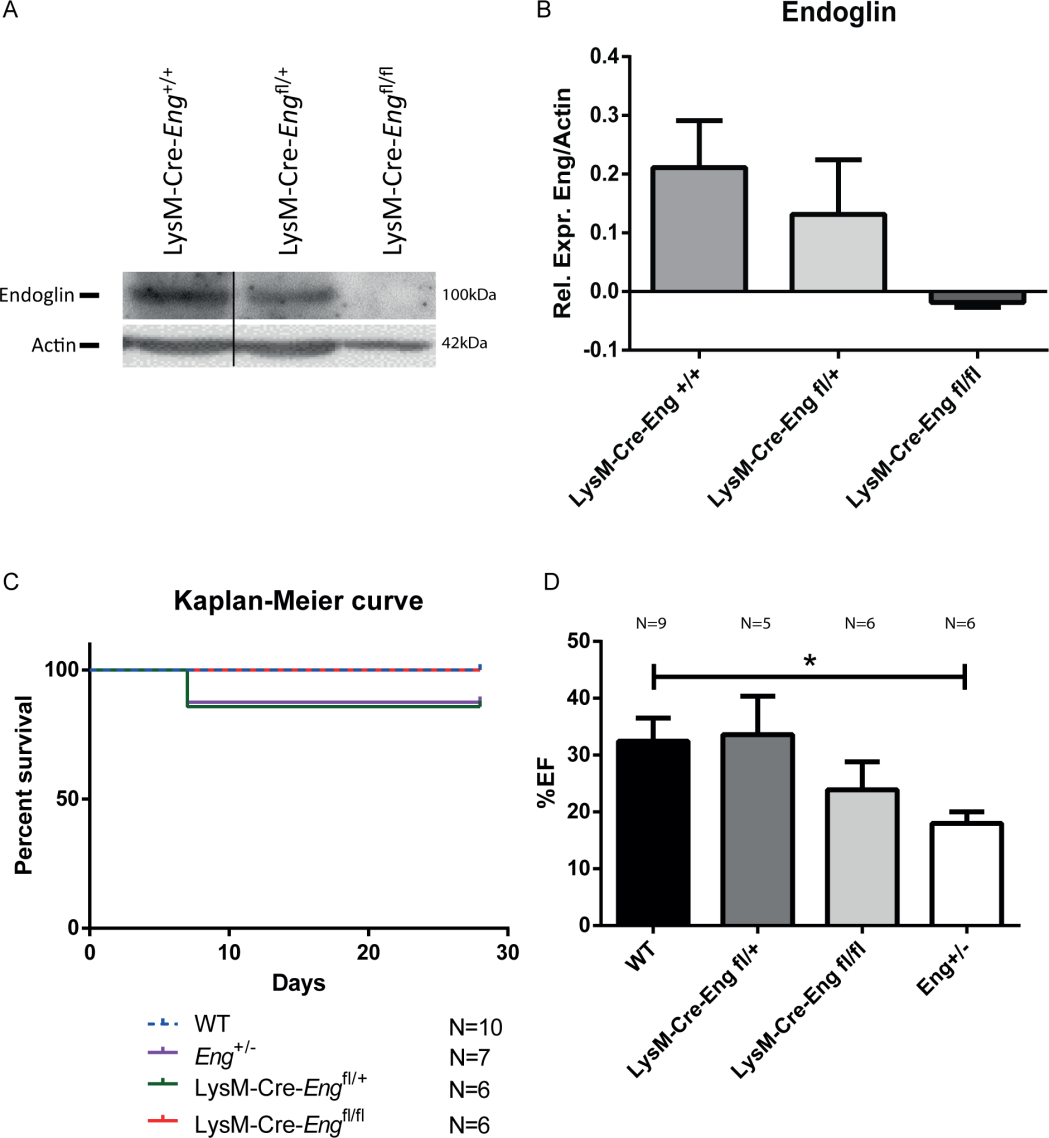
Monocyte specific knock-out of endoglin does not recapitulate the *Eng*^*+/-*^ phenotype. **(A)** Western blot analysis of endoglin protein expression in LysM-Cre-*Eng*^+/+^, LysM-Cre-*Eng*^fl/+^ and LysM-Cre-*Eng*^fl/fl^ cultured macrophages. A representative experiment is shown. **(B)** Quantification of the Western blots for endoglin protein in LysM-Cre-*Eng*^+/+^, LysM-Cre-*Eng*^fl/+^ and LysM-Cre-*Eng*^fl/fl^ cultured macrophages in two independent experiments(macrophage cultures from 3 individual mice of each genotype were pooled per western blot). **(C)** Kaplan-Meier survival curve of wild type (WT), LysM-Cre-*Eng*^fl/+^ and LysM-Cre-*Eng*^fl/fl^ mice 28 days post-MI (n = 6–10). **(D)** Cardiac function in percentage ejection fraction (%EF) 14 days post-MI. Cardiac function was measured by ultrasound in long axis view (n = 5–9). Data are shown as mean ± SEM, *P<0.05.

We have previously shown that HHT1 MNCs are impaired in their homing capacity towards ischemic tissue [14]. To establish the endogenous contribution of MNCs to the *Eng*^+/-^ phenotype, a LysM-specific mouse model for targeting myeloid cells (monocytes, macrophages and granulocytes) was generated. Mice with either a heterozygous or homozygous deletion for LysM-specific endoglin were analyzed. To validate recombination, MNCs were allowed to differentiate towards macrophages in the presence of GM-CSF and western blot analysis showed reduced endoglin expression in the LysM-Cre-*Eng*^fl/+^, and a near absence of endoglin in the LysM-Cre-*Eng*^fl/fl^ macrophages (Fig 1A and 1B).

The deletion of endoglin in monocytes did not affect survival post-MI (Fig 1C). Analysis of cardiac function showed a trend towards reduced ejection fraction (EF) in the LysM-Cre-*Eng*^fl/fl^ mice (Fig 1D, WT 34.07±3.65% vs LysM-Cre-*Eng*^fl/fl^ 22.73±4.30%, P = 0.09), suggesting an involvement of endoglin in macrophage function during cardiac repair. However, since cardiac dysfunction of the LysM-Cre-*Eng*^fl/fl^ phenotype post-MI is not as pronounced as the reduction in EF seen in the *Eng*^*+/-*^ mice after MI (Fig 1D, WT 32.45±4.06% vs. *Eng*^+/-^ 17.98±2.06%, P<0.05), we concluded that the injury response in HHT1 is likely to be an interplay between a multitude of cell types, only partially represented by the LysM population. We therefore continued by analyzing the effect of DPP4 inhibitor treatment in the *Eng*^*+/-*^ animals.

### DPP4 inhibition restores the *in vivo* MNC homing capacity in *Eng*^+/-^ mice

**Fig 2:**
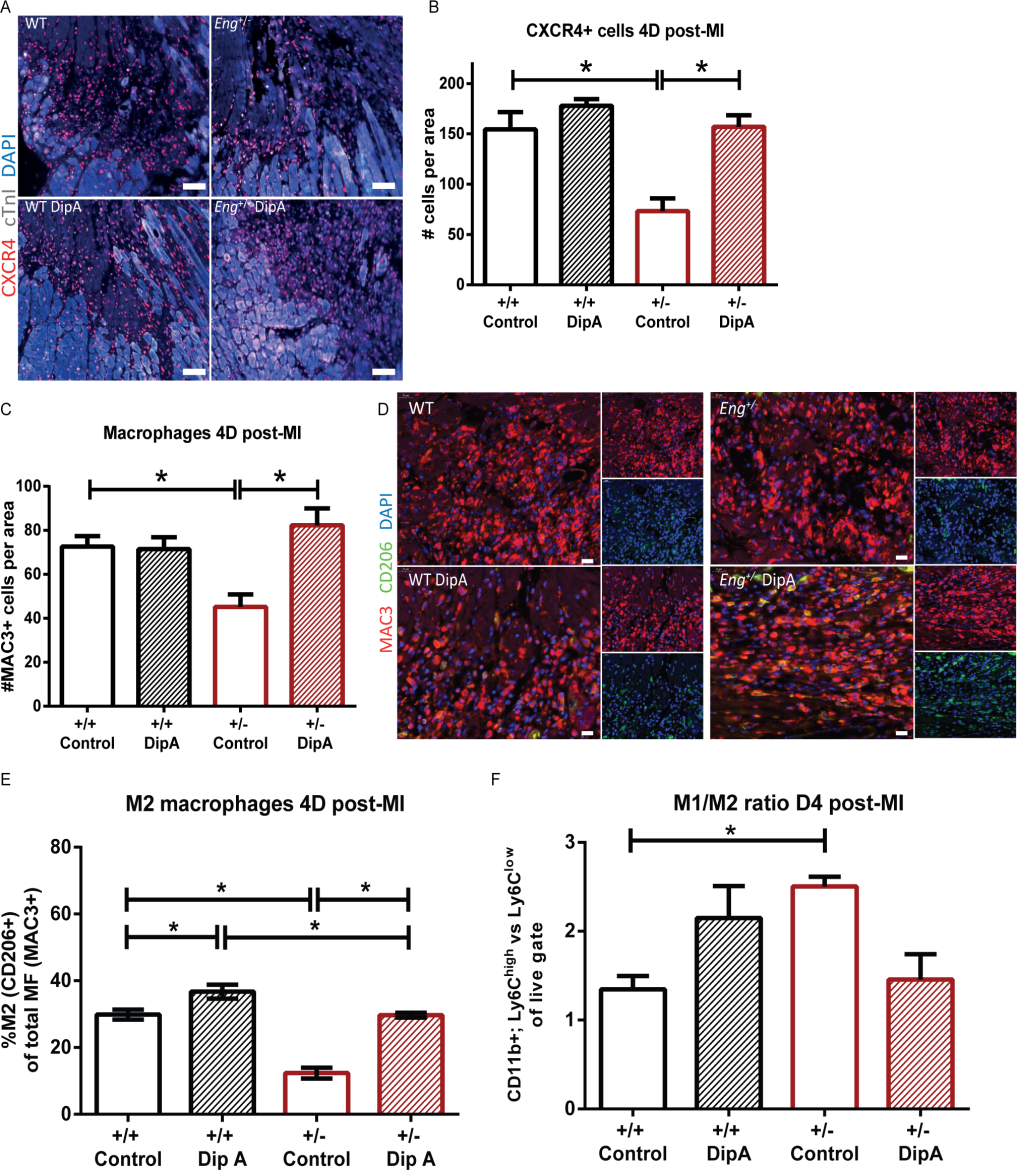
DPP4 inhibitor treatment of *Eng*^*+/-*^ mice restores homing of MNCs to injured myocardium at 4 days post-MI. **(A)** Representative microscopy images of CXCR4 expression in the infarct border zone. White = surviving myocardium, black/grey area = infarcted myocardium. Photos taken at 30x magnification. Scale bar: 50μm. CXCR4 = red, cTnI = white, DAPI nuclear staining = blue. **(B)** Quantification of CXCR4 expressing cells in the infarct border zone (n = 6–8). Data shown are mean ± SEM, *P<0.05. **(C)** Quantification of MAC3 positive cells in the infarct border zone (n = 6–7). Data shown are mean ± SEM, *P<0.05. **(D)** Representative microscopy images of MAC3^+^/CD206^-^ (%M1) and MAC3^+^/CD206^+^ (%M2) expressing cells in the infarct border zone. Smaller panels: Top panel is the MAC3 signal, lower panel is the CD206 signal. Photos taken at 50x magnification. Scale bar: 20μm MAC3 = red, CD206 = green, DAPI = blue. **(E)** Quantification of the ratio of MAC3^+^/CD206^-^ (%M1) and MAC3^+^/CD206^+^ (%M2) expressing cells in the infarct border zone (n = 6–7). **(F)** Flow cytometric analysis of the macrophage population in the infarct area, ratio of inflammatory M1(Ly6G^-^/CD11b^+^/Ly6C^high^) versus regenerative M2 macrophages (Ly6G^-^/CD11b^+^/Ly6C^low^) (n = 3–6, non-parametric ANOVA testing). Control = MQ treated, DipA = Diprotin A treated group. Data shown are mean ± SEM, *P<0.05.

Macrophage infiltration is important for proper repair of damaged tissue [12, 28, 29]. Pre-treatment of the HHT1-MNCs, expressing enhanced levels of DPP4, with a DPP4 inhibitor prior to intravenous injection after experimentally induced MI in the mouse restored homing of these cells to the infarct site. Therefore, we asked the question whether systemic administration of a DPP4 inhibitor would have the same effect, and treated mice with the DPP4 inhibitor Diprotin A (DipA) systemically via intraperitoneal (i.p.) injection from day 0 till day 5 post-MI. Flow cytometric analysis of blood samples at baseline revealed that the mice did not display any leukopenia because of endoglin heterozygosity (S1 Fig; gating strategy provided in S7A and S7B Fig). At day 4 post-MI, during the peak of inflammatory cell influx [12], MNC homing to the infarcted border zone is diminished in *Eng*^+/-^ mice as shown by a decrease in the number of CXCR4 positive MNCs in the infarct border zone (Fig 2A, quantification in B). Systemic DipA treatment of *Eng*^+/-^ mice did not affect survival up to 14 days post-MI (S2 Fig), nor did it hematopoietic cell release from the bone marrow (data not shown), or granulocyte presence in the blood or infarcted cardiac tissue (S3 Fig and gating strategy provided in S7C Fig). DipA treatment did increase the homing of CXCR4 expressing cells to the infarct border zone of *Eng*^*+/-*^ heart to similar levels as observed in MQ treated WT mice (Fig 2B, WT control 154.50±17.21 and *Eng*^*+/-*^ control 73.17±12.67 vs. *Eng*^+/-^ DipA treated 157.00±11.61, P = 0.0003). DipA treatment of control mice did not result in a significant increase in MNC influx post-MI, suggesting that a maximal homing capacity is already achieved in these animals (Fig 2B, WT control 154.50±17.21vs. WT DipA treated 178.00±6.50). The same restorative effect on homing was observed for MAC3 positive macrophages. Quantification of MAC3^+^ cells in the infarct border zone showed a decreased number of macrophages in infarcted hearts of *Eng*^+/-^ mice, but their homing is enhanced upon systemic treatment with DipA (Fig 2C, *Eng*^+/-^ 45.17±5.73 vs. *Eng*^+/-^ DipA treated 82.27±7.68, P = 0.002 and representative photos of DAB staining in S4 Fig). As observed for the CXCR4 expressing cells, DipA treatment did not affect the number of macrophages present in the infarcted hearts of wild type mice. Since there are two main populations of macrophages involved in tissue repair [12], we analyzed the effect of DPP4 inhibition on the inflammatory-like (M1) and regenerative-like (M2) macrophage subtypes in the infarct border zone (Fig 2D). Analysis of the percentage of M2 in the total macrophage population (represented by the percentage MAC3/CD206 positive cells) revealed a decrease in M2 in *Eng*^*+/-*^ mice compared to wild types (Fig 2E, WT 29.88 ±1.52% vs. *Eng*^+/-^ 12.34±1.64%, P<0.0001). After DipA treatment, there was a significant increase in the percentage of M2 macrophages within the total population of macrophages in both wild type and *Eng*^+/-^ mice, and consequently a decrease in M1 macrophages. Importantly, DipA was able to restore the M1/M2 ratio in *Eng*^+/-^ mice to WT levels. We further corroborated our findings by flow cytometric analysis of the macrophage population in the infarct area. Inflammatory M1 (Ly6G^-^/CD11b^+^/Ly6C^high^) were increased in the *Eng*^+/-^ at baseline compared to regenerative M2 macrophages (Ly6G^-^/CD11b^+^/Ly6C^low^). DPP4 inhibition showed a trend towards a decrease in M1 macrophages (Fig 2F and gating strategy provided in S7D Fig). Thus, systemic DipA treatment of *Eng*^+/-^ mice restores homing of MNCs to, as well as the macrophage M1/M2 balance in the injured heart 4 days post-MI.

### DPP4 inhibition reduces infarct size

**Fig 3:**
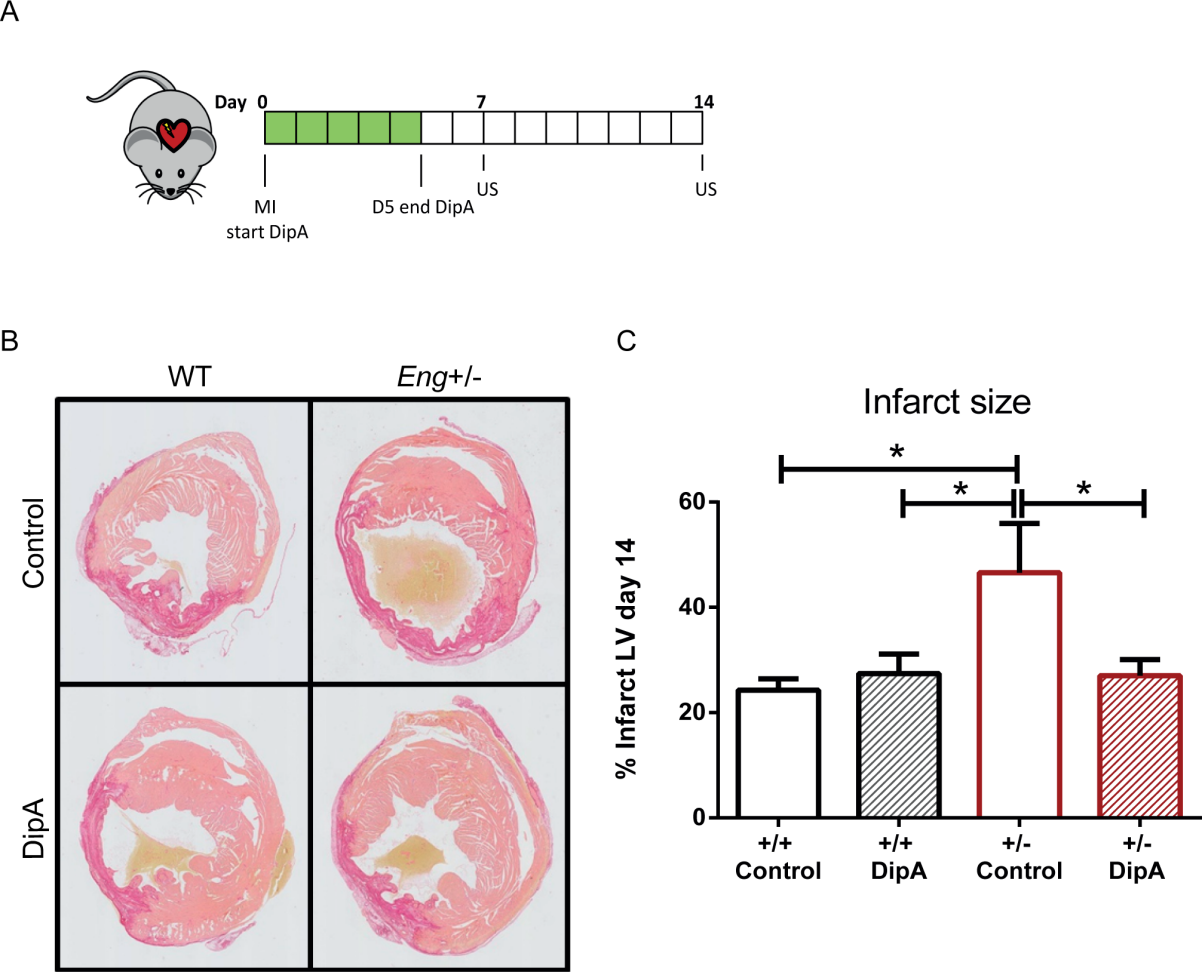
DPP4 inhibitor treatment reduces infarct size. **(A)** Experimental protocol and treatment overview. At day 0, MI is induced and DPP4 inhibition is started (shown in green) till day 5 post-MI by intraperitoneal (i.p.) injection of DipA (treatment group) or distilled water (control group). Cardiac echography was performed at day 7 and 14 post-MI. **(B)** Histological analysis of the infarct size by Picrosirius red staining for collagen (n = 5–9). Transverse sections of left ventricle, photos taken at 1.0x magnification. Infarct area = dark pink, healthy myocardium = light pink, blood cells = yellow. **(C)** Quantification of Picrosirius red staining in left ventricle (LV). Mice were subjected to MI and treated with either distilled water or DipA from day 0 till day 5 by daily i.p. injection (n = 5–9). Control = MQ treated, DipA = Diprotin A treated group. Data shown are mean ± SEM, *P<0.05.

By treating the mice with DipA from day 0 to 5 post-MI, homing was stimulated during the peak of inflammatory cell influx (Fig 3A). To assess the effect of DipA treatment on infarct size we quantified the fibrotic area using Picrosirius red staining at day 14 post-MI (Fig 3B and 3C). As expected, control *Eng*^*+/-*^ mice showed an increase in infarct size compared to WT controls (Fig 3C, WT control 24.30±2.12% vs. *Eng*^*+/-*^ control 46.60±9.33%, P = 0.009). Upon DipA treatment, no effect on infarct size was detected in wild type animals (Fig 3C, WT DipA 27.41±3.74%), however in *Eng*^*+/-*^ mice a significant decrease in infarct size was observed (Fig 3C, *Eng*^+/-^ control 46.60±9.33% vs. *Eng*^+/-^ DipA 27.02±3.04%, P = 0.02), which resulted in similar infarct sizes as observed in the wild type mice.

### Effect of DPP4 inhibition on cardiac function

**Fig 4:**
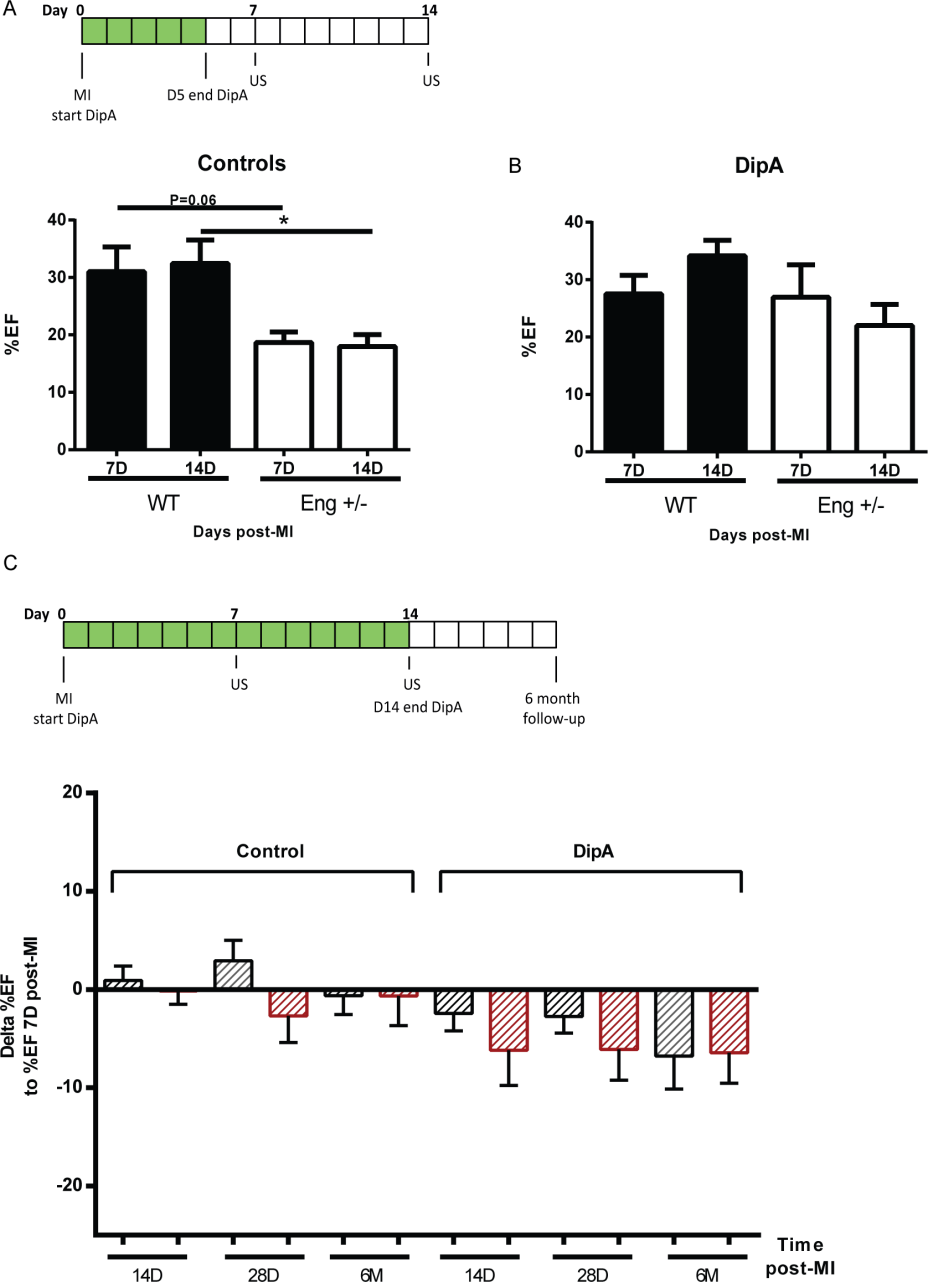
DipA treatment does not maintain improved cardiac function of *Eng*^*+/-*^ mice after MI. **(A)** Experimental overview and percentage EF at 7 and 14 days post-MI of control treated mice, measured by ultrasound via left ventricle tracing (n = 5–9). Data shown are mean ± SEM, *P<0.05. **(B)** Percentage EF 7 and 14 days post-MI of DipA treated mice, measured by ultrasound via left ventricle tracing. Note that the control WT and *Eng*^*+/-*^ groups are the repeat of measurements used for Fig 1D (n = 9–11). Data shown are mean ± SEM, *P<0.05. **(C)** Long term treatment overview and Δ%EF. DipA treatment (shown in green) up to 14 days post-MI and cardiac function with extended follow-up of 6 months (n = 5–11). Data depicted as ΔEF are the EF at the time point indicated on the x-axis compared to EF measured at day 7 post-MI. Cardiac function was measured by ultrasound via left ventricle tracing. DipA = DPP4 inhibitor Diprotin A, US = Ultrasound measurement. Control = MQ treated, DipA = Diprotin A treated group. Data shown are mean ± SEM, *P<0.05.

Since DPP4 inhibition enhanced homing of circulating MNCs to the injured heart, reduced infarct size and increased the number of regenerative macrophages present in the heart, we investigated the effect of DipA treatment on cardiac function after MI using ultrasound at baseline, and day 7 and day 14 after MI. Baseline analysis showed that cardiac function at baseline was similar between wild type and *Eng*^*+/-*^ mice (S5 Fig). At 7 and 14 days post-MI, control treated *Eng*^+/-^ animals showed a significantly lower cardiac EF compared to control treated wild type mice (Fig 4A, WT control day 7; 31.01±4.32% vs. *Eng*^+/-^ control day 7;18.68±1.84% and WT control day 14; 32.45±4.06% vs. *Eng*^+/-^ control day 14; 17.98±2.06%). Treating wildtype animals with DipA did not result in significant changes in the EF (Fig 4B, also compare to 4A, WT DipA treated day 7; 27.53±3.24% vs. WT DipA treated day 14; 34.15±2.69%). Interestingly, DipA treatment in *Eng*^+/-^ did result in normalization of the EF to the same values as WT animals at day 7 (Fig 4B, *Eng*^+/-^ DipA treated day 7; 26.94±5.65%), although at day 14, it was less pronounced (Fig 4B, *Eng*^+/-^ DipA treated day 14; 22.02±3.65%). Nevertheless, these functional data coincide with the reduced infarct size observed in Fig 3.

As we observed that endoglin heterozygosity skews the M1/M2 ratio towards a more inflammatory profile, both homing and/or differentiation of *Eng*^*+/-*^ MNCs might be delayed. Therefore, we prolonged the daily DipA treatment from 5 till 14 days post-MI. Extended DPP4 inhibition resulted in a similar decrease in cardiac function in wild type mice compared to short term (5 days post-MI) treated mice (Fig 4C). While initially DPP4 inhibition in the *Eng*^+/-^ animals improves cardiac function, *Eng*^+/-^ mice showed a gradual reduction of cardiac function in the 6 month follow-up (ΔEF in Fig 4C and %EF is provided in S6 Fig). Thus, neither short term nor prolonged DipA treatment resulted in a long term beneficial effect on heart function in *Eng*^+/-^ mice.

### DPP4 inhibition increases angiogenesis but decreases vessel maturation in *Eng*^+/-^ animals

**Fig 5:**
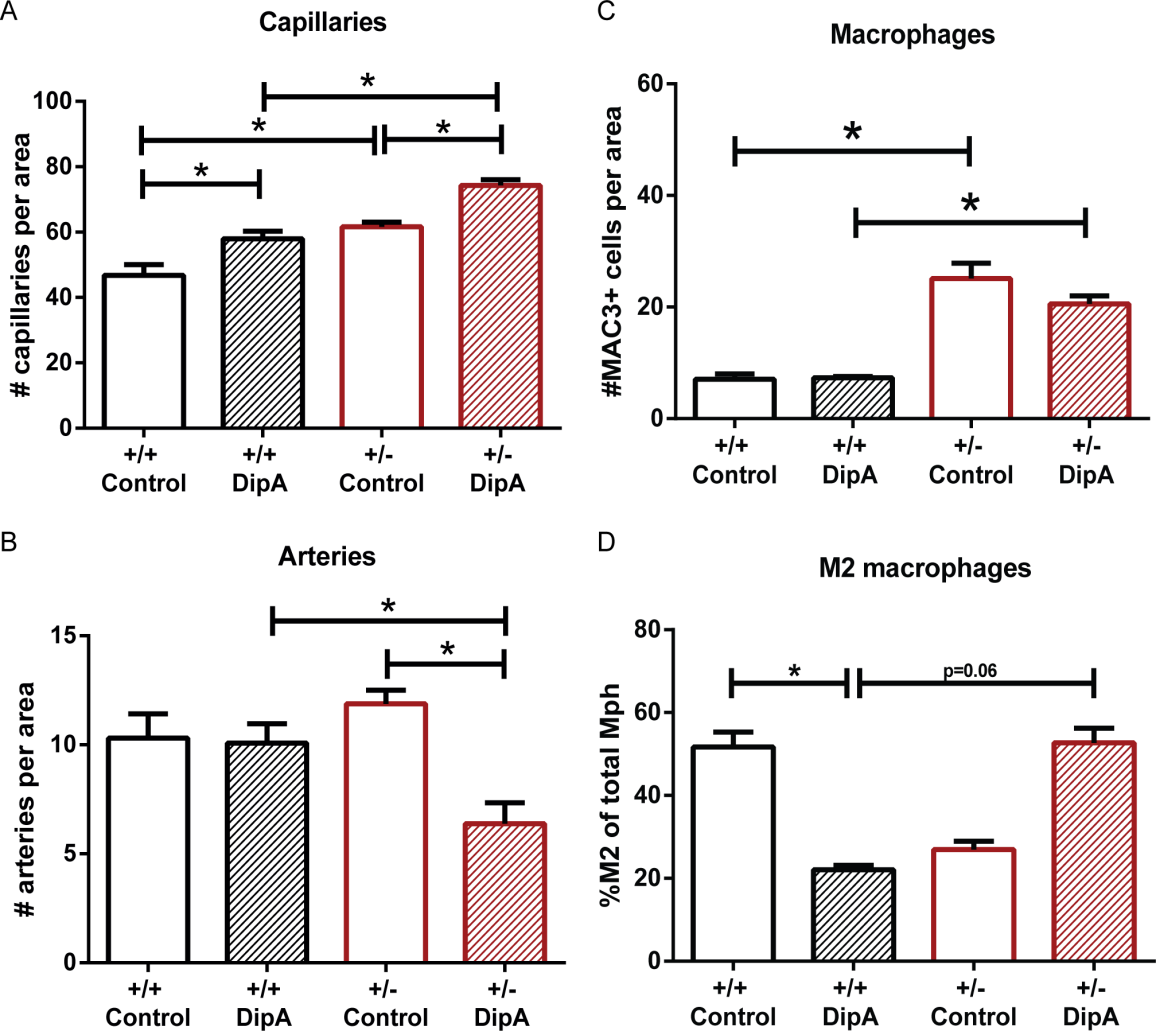
DPP4 inhibition affects angiogenesis in the infarct border zone post-MI. **(A)** Capillaries in infarct border zone, day 14 post-MI. Quantification of PECAM-1 positive vessels per area (n = 4–6). Data shown are mean ± SEM, *P<0.05. **(B)** Arteries in infarct border zone, day 14 post-MI. Quantification of PECAM-1/αSMA positive vessels per area (n = 4–6). Data shown are mean ± SEM, *P<0.05. **(C)** Quantification of total macrophage (MAC3) number in day 14 post-MI hearts (n = 4–6). Data shown are mean ± SEM, *P<0.05. **(D)** Quantification of MAC3^+^/CD206^+^ (%M2) expressing cells in the infarct border zone 14 days post-MI (n = 3–6). Control = MQ treated, DipA = Diprotin A treated group. Data shown are mean ± SEM, *P<0.05.

Since neoangiogenesis is important for cardiac repair post-MI, we investigated the effect of DipA treatment on vascularization by staining the hearts for PECAM-1 and αSMA and analyzed the presence of capillaries and arteries in the infarct border zone. The number of capillaries in the *Eng*^+/-^ mice was increased compared to wild type animals (Fig 5A). In both wild type as well as *Eng*^+/-^ mice, DipA treatment further increased the number of capillaries present in the border zone (Fig 5A, *Eng*^+/-^ 61.63±1.43 vs. *Eng*^+/-^ treated 74.30±1.74, P = 0.001). Surprisingly, the number of arteries in the infarct border zone was significantly lower in the DPP4 inhibitor treated *Eng*^*+/-*^ mice (*Eng*^+/-^ 11.88±0.63 vs. *Eng*^+/-^ treated 6.38±0.97, P = 0.003), which may suggest that vessel maturation or arteriogenesis is impaired (Fig 5B).

### Macrophage presence in the ventricular wall is prolonged in *Eng*^+/-^ mice

Tissue repair is a tightly controlled process, with a delicate balance between the influx of inflammatory cells needed to remove cell debris after ischemia, and their resolution thereafter when damaged tissue is repaired. A prolongation of the inflammatory phase is detrimental for tissue repair. Interestingly, a significantly higher number of macrophages was observed in the infarct border zone of the *Eng*^+/-^ mice compared to the wild type mice 14 days post-MI (Fig 5C, WT 7.06±0.97 vs. *Eng*^+/-^ 25.13±2.76, P<0.0001). DipA treatment of *Eng*^+/-^ did not reduce the total number of macrophages to wild type levels.

Analyzing the macrophage phenotypes, we observed a disbalance in the M1/M2 ratio in the hearts of *Eng*^+/-^ mice 4 days post-MI. Therefore, macrophage quantity and subtype in the heart was also analyzed 14 days post-MI. Interestingly, *Eng*^+/-^ DipA treated mice showed an increase in M2 levels reaching wild type levels (Fig 5D WT 51.7% vs. *Eng*^+/-^ treated 52.7%), while in wild type mice DipA treatment resulted in a significant drop in the percentage of M2 cells (Fig 5D WT 51.7% vs. WT treated 22.0%, P = 0.04). These results suggest that there is a prolonged inflammatory response and possibly an impaired resolution of inflammation in *Eng*^+/-^ mice, and the macrophage balance can be skewed towards the M2 subtype by DPP4 inhibition.

## Discussion

In this study we demonstrate that after MI, the decrease in myocardial function observed in our HHT1 animal model is not solely caused by a disturbed endothelium response, but also in part by impaired MNC function. MNC homing and differentiation into inflammatory and regenerative macrophages is important for proper tissue repair. In our previous research, we found that MNC homing towards the ischemic myocardium is severely affected by endoglin heterozygosity, together with enhanced DPP4 levels [15]. Here, we demonstrate that homing of MNCs to the ischemic myocardium could be restored by systemic DPP4 inhibition. Although DipA treatment did reduce the infarct size in *Eng*^+/-^ mice to similar levels as in wild type animals, it did not result in long term improvement of EF. Detailed analysis of the infarcted ventricle demonstrated that neovascularization was increased in the *Eng*^+/-^ infarct border zone upon DPP4 inhibition, while arteriogenesis or vessel maturation was dramatically decreased. Furthermore, analyzing the macrophage subsets, a prolonged presence of inflammatory-like macrophages (M1) was observed. We therefore conclude that the inflammatory and regenerative function of *Eng*^+/-^ macrophages is affected. Interestingly, DPP4 inhibition could restore this M1/M2 imbalance to WT levels.

Complete endoglin deficiency in mice results in embryonic lethality around mid-gestation due to cardiovascular defects and the formation of enlarged, fragile vessels [4]. *Eng*^+/-^ mice or endothelial deletion of endoglin in adult mice results in a relatively mild phenotype. Only after a secondary hit like induction of tissue damage or inflammation, the impairments of endoglin deficiency become apparent in the form of reduced angiogenesis and the development of AVMs [30–32]. In the current study, we observed that the macrophage specific deletion of *Eng* did not result in similar levels of cardiac deterioration as found in *Eng*^+/-^ animals, implying a multifactorial nature of HHT1. This emphasizes the need for endoglin heterozygosity to be present in all cell types of the heart to recapitulate the HHT1 disease phenotype.

Furthermore, we show that the homing capacity of MNCs in the *Eng*^+/-^ mice could be restored by systemic DPP4 inhibitor treatment. In cardiovascular patients an association was found between increased DPP4 presence on MNCs and poor recovery after coronary intervention [33]. Together with our observation of decreased homing capacity of the MNCs, HHT1 now proves to affect other cell types and functions as well, in particular the lymphocytes and monocytes [31, 32, 34, 35]. Macrophages are an important subfraction of the MNC population, as macrophages have both inflammatory and angiogenic capacity and are essential in tissue regeneration and remodeling [28]. The shift of the inflammatory-like macrophages (M1) to the regenerative-like macrophages (M2) is essential for optimal resolution of inflammation [36]. Immune function and the angiogenic capacity of macrophages are most likely impaired in *Eng*^+/-^ mice [37, 38]. Recent studies suggested that there is an intrinsic inflammatory defect in HHT1 patients as well. Investigators report leukopenia, increased risk of infection [34] and impaired endoglin upregulation in activated monocytes of HHT1 patients [35]. We now observed an increased ratio of M1/M2 macrophages in *Eng*^+/-^ mice hearts post-MI, which may indicate that the increase in M1 macrophages is due to either impaired differentiation of the M1 towards M2 macrophages, or decreased homing capacity of M2 macrophages. In renal ischemia-reperfusion injury, the same disbalance in M1/M2 was also reported in *Eng*^+/-^ mice [39]. DPP4 inhibition has been shown to shift the M1/M2 balance towards more reparative macrophages [22, 23]. We show that in *Eng*^+/-^ mice DipA treatment restored both the total number of macrophages as well as the M1/M2 ratio. This effect was maintained up to 10 days after cessation of DPP4 inhibitor treatment. The shift of the macrophage population towards M2 induced by DPP4 inhibition was also observed by Brenner et al. [22], when analyzing macrophage presence in the aortic walls of *ApoE*^-/-^ mice in a model for atherosclerosis. Likewise, a reduction of atherosclerotic lesions [20] and total macrophage presence was reported in *ApoE*^-/-^ mice treated with DPP4 inhibitor, together with a reduction in smooth muscle cell proliferation [40]. DPP4-null mice had an increased expression of M2 genes [41] and furthermore, DPP4 inhibition had anti-inflammatory effects on macrophages in general by decreasing pro-inflammatory NFκB signaling [42]. An increase in M2 macrophages was also observed upon DPP4 inhibitor treatment in another atherosclerosis model using *LDLR*^-/-^ mice [43], moreover, a recent meta-analysis showed a decreased infection rate in T2DM patients treated with a DPP4 inhibitor [44]. Here we show for the first time, to our knowledge, that DPP4 inhibition polarizes the M1/M2 ratio towards a more regenerative phenotype in a mouse model for HHT1. Furthermore, our data suggests that endoglin heterozygosity not only impairs homing of the MNCs, but affects proper differentiation and/or function of the macrophages, as we find more M1 at the infarct site and a decrease in resolution of immune cells 14 days post-MI in *Eng*^+/-^ animals.

In addition to improved homing of circulating (stem) cells, we and others show that DPP4 inhibition significantly stimulated vascularization and cardiac repair [45–47]. In conjunction, an increase in capillary number was observed at the infarct border zone, confirming an increase in endothelial regeneration, possibly by enhanced presence of regenerative M2 macrophages. Interestingly, the number of arteries present was found to be significantly lowered in the DPP4 inhibitor treated *Eng*^+/-^ mice. Vessel maturation is known to be affected in *Eng*^+/-^ mice, due to dysfunctional mural cells and pericytes [48, 49]. Our results therefore suggest that DPP4 inhibitor treatment most likely further impairs vessel maturation in *Eng*^+/-^ mice, due to its involvement in vascular smooth muscle cell recruitment [50, 51].

DPP4 inhibitors have been reported to increase cardiomyocyte survival, however the exact mechanism is not yet fully understood. DPP4 has many targets besides SDF1, like atrial natriuretic peptide (ANP)- a protein associated with cardiomyocyte survival [52]. DPP4 inhibition might therefore decrease the inactivation of ANP by DPP4, and contribute to cardiomyocyte survival/protection. Furthermore, when MI was performed in DPP4^-/-^ mice, these mice show an increased survival compared to DPP4^+/+^ mice [52]. DPP4 inhibitor treatment in previous reports already showed a modest, albeit not significant gain of cardiac function in wild type mice after MI [53]. Interestingly, at day 14 post-MI the infarct size in *Eng*^+/-^ mice treated with DipA was significantly smaller and similar in size compared to wild type control levels. Concordantly, we observed an initial increase in EF upon DipA treatment. However, improved cardiac function in *Eng*^+/-^ mice was not preserved at day 14 post-MI. These results suggest that DPP4 inhibition conveys a decrease of the fibrotic response in *Eng*^+/-^ animals, resulting in a reduced infarct size, though this is not reflected in a sustained improvement of heart function. This could be in part explained by the fact that although capillary presence is increased, in HHT1 endothelial functionality is impaired, forming leaky vessels. Secondly, the misbalanced MNC population present in HHT1 might result in an exaggerated and prolonged immune response, which is likely to disturb tissue repair.

We hypothesized that when homing of *Eng*^+/-^ MNCs to the infarct site was restored, cardiac function would improve, as was observed in wild type animals [46, 53]. Although increased homing and a decrease in infarct size were observed, this did not correlate with a long term positive effect on cardiac function. The *Eng*^+/-^ mice showed either a reduced differentiation towards, or presence of the regenerative macrophage subtype and interestingly this level could be corrected by DPP4 inhibition. However, although the fibrotic response was restored to wild type levels and MNC recruitment was re-established, the *Eng*^+/-^ mice still show a decrease in cardiac function. This suggests that the impaired function of the *Eng*^+/-^ MNCs that homed to the infarct site still exists. Combining DPP4 inhibition with G-CSF treatment for enhanced MNC recruitment, Zaruba et al. showed that ameliorated MNC homing resulted in a significant increase in cardiac function in mice. However, testing this treatment in patients suffering from MI, the combination failed to show any beneficial effect on cardiac function [54, 55].

How DPP4 inhibition affects MNC function is still unclear. DPP4 inhibition is already being tested in several clinical trials in cardiovascular disease (CVD), and meta-analyses of the risks involved have proven to be varied in outcome. Several meta-analyses have been performed in the field of T2DM. Upon DPP4 inhibition, no increase in CVD was observed in T2DM patients, but risk of infection was reduced [44]. In addition, although no effect was seen on cardiovascular mortality, short term DPP4 inhibition reduced chances of MI events, while long term treatment showed a possible increase in heart failure cases [56, 57]. Other meta-analyses showed either no effect or a reduction of cardiovascular events, also with long term treatment [58–61].

As described in this study, DPP4 inhibition positively affects cardiac fibrosis and initially, also cardiac function. We showed by using a PSR staining that fibrosis is enhanced in *Eng*^*+/-*^ mice and decreases upon DPP4 inhibition. A relation between DPP4 inhibition and reduction in fibrosis was also demonstrated in a kidney model [62]. Furthermore, inhibition of DPP4-positive fibroblasts reduced scarring of murine dermal wounds [63] and DPP4 inhibition also mediated antifibrotic effects in dermal fibroblasts [64]. DPP4 inhibition is shown to be cardioprotective in several studies (reviewed in Grilo et al. [65]). A study overexpressing DPP4 resulted in promoted mammary tumorigenesis and transformation of epithelial cells [66], altogether indicating DPP4 inhibition is the way forward.

DPP4 and its inhibition has a multitude of functions and effects, many of which are still poorly understood. This is exemplified by the study from Zhu et al. [67], where they show that DPP4 is able to target neuropeptide Y and peptide YY in the cardiac nerves, proteins that can result in activation of cardiac fibroblasts. To the contrary, many anti-fibrotic aspects have been described, and by various mechanisms; DPP4 inhibitors were reported to lower active TGFβ (via reduction of DPP4 -CIM6PR membrane receptor interaction) and also decrease fibronectin expression [68–70]. Together, the beneficial effects of DPP4 inhibition on MNC homing, reduction in fibrosis and restoration of regenerative macrophage differentiation could provide a suitable treatment for general tissue repair in HHT1 patients. However, this study suggests that treating HHT1 patients with a DPP4 inhibitor post-MI should not be considered as monotherapy, but ought to be combined with additional MNC stimulating agents and/or arteriogenic stimulation. Although DPP4 inhibition is an accepted treatment in T2DM patients, a better understanding of its mode of action should first be gained before further conducting clinical trials for applying this treatment on any other disease or disorder like HHT1.

## Supporting information

S1 Fig *Eng*^*+/-*^ mice do not display leukopenia. Flow cytometric analysis of the major leukocyte subsets in the circulation of the mice groups at day 0 (pre-MI and pre-DipA treatment). Leukocytes labeled for anti-mouse CD3, CD3^+^/CD4^+^, CD3^+^/CD8^+^ and CD11b^+^/Ly6G^-^ (n = 3–6, non-parametric ANOVA testing). Control = MQ treated, DipA = Diprotin A treated group. Data shown are mean ± SEM, *P<0.05.
(TIF)

S2 Fig DPP4 inhibition does not affect survival of WT and *Eng*^+/-^ mice. Kaplan-Meier curve of WT and *Eng*^+/-^ mice 14 days post-MI. Graph depicts percentage of surviving WT and *Eng*^+/-^ mice, control and DipA treated animals (n = 12–18). Control = MQ treated, DipA = Diprotin A treated group.
(TIF)

S3 Fig The granulocyte subset is not affected in *Eng*^+/-^ mice at baseline or by DipA treatment 4 days post-MI. Flow cytometric analysis of the granulocyte subset in the circulation of the mice at **(A)** day 0 (pre-MI and pre-DipA treatment) and **(B)** day 2 and **(C)** 4 post-MI in the circulation. **(D)** Granulocytes isolated from the infarcted part of the LV 4 days post-MI. Leukocytes labeled with anti-mouse CD11b, Ly6G. Granulocytes were identified as the CD11b^-^/Ly6G^+^ population of the live gate (n = 3–6, non-parametric ANOVA testing). Control = MQ treated, DipA = Diprotin A treated group. Data shown are mean ± SEM, *P<0.05.
(TIF)

S4 Fig MAC3 expressing cells in the infarct border zone 4 days post-MI. Transversal sections of mouse hearts were stained for macrophage marker MAC3 using immunohistochemistry (n = 6–7). Photos taken at 15x magnification. MAC3 = brown, nuclei = blue.
(TIF)

S5 Fig *Eng*^*+/-*^ mice show no difference in baseline ejection fraction. Baseline cardiac function in percent ejection fraction (%EF) between WT and *Eng*^*+/-*^ mice (n = 4). Cardiac function was measured by ultrasound. Data shown are mean ± SEM, *P<0.05.
(TIF)

S6 Fig DPP4 inhibition does not improve cardiac function long term. Percentage EF 7, 14, 28 days and 6 months post-MI of WT(+/+) and *Eng*^+/-^ (+/-) mice, control (Milli-Q ultrapure sterile water, MQ) and DipA treated animals. EF was measured by ultrasound and analyzed by left ventricle tracing (n = 5–11). Data shown are mean ± SEM, *P<0.05.
(TIF)

S7 Fig Flow cytometry gating strategy T-cells, monocytes and macrophages. **(A)** Gating strategy for CD3, 4 and 8 T-cells in the blood. The first gating step is a gate for live cells using FSC and SSC. T-cells are subsequently identified using CD3. T-cells subsets are then identified with CD4 and CD8.
**(B)** Monocytes gating strategy in the blood. The first gating step is a gate for live cells using FSC and SSC. The second gating step is for monocytes, identified by CD11b positive and Ly6G negative labeling.
**(C)** Granulocyte gating strategy in the blood. The first gating step is a gate for live cells using FSC and SSC, the granulocytes are subsequently identified by Ly6G.
**(D)** Macrophage subsets gating strategy from MNCs isolated from the left ventricle. The first gating step is a gate for live cells using FSC and SSC, the monocytes are identified by CD11b positive and Ly6G negative labeling. The inflammatory-like M1 macrophages are then subsequently identified by Ly6C^high^ and regenerative-like M2 macrophages identified by Ly6C^low^ labeling.
(TIF)

## References

[1] Lebrin F, Goumans M-J, Jonker L, Carvalho RLC, Valdimarsdottir G, Thorikay M, et al Endoglin promotes endothelial cell proliferation and TGF-β/ALK1 signal transduction. The EMBO Journal. 2004;23:4018–28. doi: 10.1038/sj.emboj.7600386 . 15385967 10.1038/sj.emboj.7600386 PMC524335

[2] Goumans M-J, Lebrin F, Valdimarsdottir G. Controlling the Angiogenic SwitchA Balance between Two Distinct TGF-b Receptor Signaling Pathways. Trends in Cardiovascular Medicine. 2003;13:301–7. doi: 10.1016/S1050-1738(03)00142-7 14522471 10.1016/s1050-1738(03)00142-7

[3] Bourdeau A, Dumont DJ, Letarte M. A murine model of hereditary hemorrhagic telangiectasia. Journal of Clinical Investigation. 1999;104:1343–51. doi: 10.1172/JCI8088 10562296 10.1172/JCI8088 PMC409846

[4] Arthur HM, Ure J, Smith AJH, Renforth G, Wilson DI, Torsney E, et al Endoglin, an Ancillary TGFβ Receptor, Is Required for Extraembryonic Angiogenesis and Plays a Key Role in Heart Development. Developmental Biology. 2000;217:42–53. doi: 10.1006/dbio.1999.9534 . 10625534 10.1006/dbio.1999.9534

[5] Bourdeau A, Faughnan ME, McDonald M-L, Paterson AD, Wanless IR, Letarte M. Potential Role of Modifier Genes Influencing Transforming Growth Factor-β1 Levels in the Development of Vascular Defects in Endoglin Heterozygous Mice with Hereditary Hemorrhagic Telangiectasia. The American Journal of Pathology. 2001;158:2011–20. doi: 10.1016/S0002-9440(10)64673-1 . 11395379 10.1016/s0002-9440(10)64673-1 PMC1891990

[6] Jerkic M, Rodríguez-Barbero A, Prieto M, Toporsian M, Pericacho M, Rivas-Elena JV, et al Reduced angiogenic responses in adult Endoglin heterozygous mice. Cardiovascular research. 2006;69:845–54. doi: 10.1016/j.cardiores.2005.11.020 . 16405930 10.1016/j.cardiores.2005.11.020

[7] Seghers L, de Vries MR, Pardali E, Hoefer IE, Hierck BP, Dijke Pt, et al Shear induced collateral artery growth modulated by endoglin but not by ALK1. Journal of Cellular and Molecular Medicine. 2012;16:2440–50. doi: 10.1111/j.1582-4934.2012.01561.x . 22436015 10.1111/j.1582-4934.2012.01561.x PMC3823438

[8] López-novoa JM, Bernabeu C, Castonguay R, Werner ED, Matthews RG, Presman E, et al The physiological role of endoglin in the cardiovascular system. American Journal of Physiology-Heart and Circulatory Physiology. 2010;299:959–74. doi: 10.1152/ajpheart.01251.2009 20656886 10.1152/ajpheart.01251.2009

[9] Liu Z, Lebrin F, Maring JA, van den Driesche S, van der Brink S, van Dinther M, et al ENDOGLIN Is Dispensable for Vasculogenesis, but Required for Vascular Endothelial Growth Factor-Induced Angiogenesis. PLoS ONE. 2014;9:e86273 doi: 10.1371/journal.pone.0086273 24489709 10.1371/journal.pone.0086273 PMC3904881

[10] Kapur N, Morine K, Letarte M. Endoglin: a critical mediator of cardiovascular health. Vascular Health and Risk Management. 2013;9:195 doi: 10.2147/VHRM.S29144 23662065 10.2147/VHRM.S29144 PMC3647444

[11] Ben-Mordechai T, Holbova R, Landa-Rouben N, Harel-Adar T, Feinberg MS, Abd Elrahman I, et al Macrophage subpopulations are essential for infarct repair with and without stem cell therapy. Journal of the American College of Cardiology. 2013;62:1890–901. doi: 10.1016/j.jacc.2013.07.057 . 23973704 10.1016/j.jacc.2013.07.057

[12] Nahrendorf M, Swirski FK, Aikawa E, Stangenberg L, Wurdinger T, Figueiredo J-L, et al The healing myocardium sequentially mobilizes two monocyte subsets with divergent and complementary functions. The Journal of experimental medicine. 2007;204:3037–47. doi: 10.1084/jem.20070885 . 18025128 10.1084/jem.20070885 PMC2118517

[13] van Amerongen MJ, Harmsen MC, van Rooijen N, Petersen AH, van Luyn MJA. Macrophage depletion impairs wound healing and increases left ventricular remodeling after myocardial injury in mice. The American journal of pathology. 2007;170:818–29. doi: 10.2353/ajpath.2007.060547 . 17322368 10.2353/ajpath.2007.060547 PMC1864893

[14] van Laake LW, van den Driesche S, Post S, Feijen A, Jansen MA, Driessens MH, et al Endoglin Has a Crucial Role in Blood Cell-Mediated Vascular Repair. Circulation. 2006;114:2288–97. doi: 10.1161/CIRCULATIONAHA.106.639161 . 17088457 10.1161/CIRCULATIONAHA.106.639161

[15] Post S, Smits AM, van den Broek AJ, Sluijter JPG, Hoefer IE, Janssen BJ, et al Impaired recruitment of HHT-1 mononuclear cells to the ischaemic heart is due to an altered CXCR4/CD26 balance. Cardiovascular Research. 2010;85:494–502. doi: 10.1093/cvr/cvp313 . 19762327 10.1093/cvr/cvp313

[16] Askari AT, Unzek S, Popovic ZB, Goldman CK, Forudi F, Kiedrowski M, et al Effect of stromal-cell-derived factor 1 on stem-cell homing and tissue regeneration in ischaemic cardiomyopathy. Lancet. 2003;362:697–703. doi: 10.1016/S0140-6736(03)14232-8 . 12957092 10.1016/S0140-6736(03)14232-8

[17] Ceradini DJ, Kulkarni AR, Callaghan MJ, Tepper OM, Bastidas N, Kleinman ME, et al Progenitor cell trafficking is regulated by hypoxic gradients through HIF-1 induction of SDF-1. Nature medicine. 2004;10:858–64. doi: 10.1038/nm1075 . 15235597 10.1038/nm1075

[18] Read PA, Khan FZ, Heck PM, Hoole SP, Dutka DP. DPP-4 Inhibition by Sitagliptin Improves the Myocardial Response to Dobutamine Stress and Mitigates Stunning in a Pilot Study of Patients With Coronary Artery Disease. Circulation: Cardiovascular Imaging. 2010;3:195–201. doi: 10.1161/CIRCIMAGING.109.899377 . 20075143 10.1161/CIRCIMAGING.109.899377

[19] Best JH, Hoogwerf BJ, Herman WH, Pelletier EM, Smith DB, Wenten M, et al Risk of cardiovascular disease events in patients with type 2 diabetes prescribed the glucagon-like peptide 1 (GLP-1) receptor agonist exenatide twice daily or other glucose-lowering therapies: a retrospective analysis of the LifeLink database. Diabetes care. 2011;34:90–5. doi: 10.2337/dc10-1393 . 20929995 10.2337/dc10-1393 PMC3005487

[20] Ta NN, Schuyler CA, Li Y, Lopes-Virella MF, Huang Y. DPP-4 (CD26) Inhibitor Alogliptin Inhibits Atherosclerosis in Diabetic Apolipoprotein E–Deficient Mice. Journal of Cardiovascular Pharmacology. 2011;58:157–66. doi: 10.1097/FJC.0b013e31821e5626 21558879 10.1097/FJC.0b013e31821e5626 PMC3155015

[21] Fadini GP, Boscaro E, Albiero M, Menegazzo L, Frison V, de Kreutzenberg S, et al The Oral Dipeptidyl Peptidase-4 Inhibitor Sitagliptin Increases Circulating Endothelial Progenitor Cells in Patients With Type 2 Diabetes: Possible role of stromal-derived factor-1. Diabetes Care. 2010;33:1607–9. doi: 10.2337/dc10-0187 . 20357375 10.2337/dc10-0187 PMC2890368

[22] Brenner C, Franz WM, Kühlenthal S, Kuschnerus K, Remm F, Gross L, et al DPP-4 inhibition ameliorates atherosclerosis by priming monocytes into M2 macrophages. International Journal of Cardiology. 2015;199:163–9. doi: 10.1016/j.ijcard.2015.07.044 26197403 10.1016/j.ijcard.2015.07.044

[23] Waumans Y, Vliegen G, Maes L, Rombouts M, Declerck K, Van Der Veken P, et al The Dipeptidyl Peptidases 4, 8, and 9 in Mouse Monocytes and Macrophages: DPP8/9 Inhibition Attenuates M1 Macrophage Activation in Mice. Inflammation. 2016;39:413–24. doi: 10.1007/s10753-015-0263-5 26454447 10.1007/s10753-015-0263-5

[24] Choi E-J, Chen W, Jun K, Arthur HM, Young WL, Su H. Novel brain arteriovenous malformation mouse models for type 1 hereditary hemorrhagic telangiectasia. PloS one. 2014;9:e88511 doi: 10.1371/journal.pone.0088511 . 24520391 10.1371/journal.pone.0088511 PMC3919779

[25] Clausen BE, Burkhardt C, Reith W, Renkawitz R, Förster I. Conditional gene targeting in macrophages and granulocytes using LysMcre mice. Transgenic Research. 1999;8:265–77. doi: 10.1023/A:1008942828960 10621974 10.1023/a:1008942828960

[26] Duim SN, Kurakula K, Goumans MJ, Kruithof BPT. Cardiac endothelial cells express Wilms' tumor-1. Wt1 expression in the developing, adult and infarcted heart. Journal of Molecular and Cellular Cardiology. 2015;81:127–35. doi: 10.1016/j.yjmcc.2015.02.007 . 25681586 10.1016/j.yjmcc.2015.02.007

[27] Sager HB, Hulsmans M, Lavine KJ, Moreira MB, Heidt T, Courties G, et al Proliferation and Recruitment Contribute to Myocardial Macrophage Expansion in Chronic Heart Failure. Circulation Research. 2016;119:853–64. doi: 10.1161/CIRCRESAHA.116.309001 . 27444755 10.1161/CIRCRESAHA.116.309001 PMC5378496

[28] Mantovani A, Biswas SK, Galdiero MR, Sica A, Locati M. Macrophage plasticity and polarization in tissue repair and remodelling. The Journal of pathology. 2013;229:176–85. doi: 10.1002/path.4133 . 23096265 10.1002/path.4133

[29] Frantz S, Nahrendorf M. Cardiac macrophages and their role in ischemic heart disease. Cardiovascular research. 2014;102:1–9. doi: 10.1093/cvr/cvu025 . 24501331 10.1093/cvr/cvu025 PMC3989449

[30] Garrido-Martin EM, Nguyen H-L, Cunningham Ta, Choe S-W, Jiang Z, Arthur HM, et al Common and distinctive pathogenetic features of arteriovenous malformations in hereditary hemorrhagic telangiectasia 1 and hereditary hemorrhagic telangiectasia 2 animal models—brief report. Arteriosclerosis, thrombosis, and vascular biology. 2014;34:2232–6. doi: 10.1161/ATVBAHA.114.303984 . 25082229 10.1161/ATVBAHA.114.303984

[31] Rossi E, Sanz-Rodriguez F, Eleno N, Düwell A, Blanco FJ, Langa C, et al Endothelial endoglin is involved in inflammation: Role in leukocyte adhesion and transmigration. Blood. 2013;121:403–15. doi: 10.1182/blood-2012-06-435347 . 23074273 10.1182/blood-2012-06-435347

[32] Peter MR, Jerkic M, Sotov V, Douda DN, Ardelean DS, Ghamami N, et al Impaired Resolution of Inflammation in the Endoglin Heterozygous Mouse Model of Chronic Colitis. Mediators of Inflammation. 2014;2014:1–13. doi: 10.1155/2014/767185 25114380 10.1155/2014/767185 PMC4121192

[33] Post S, van den Broek AJ, Rensing BJ, Pasterkamp G, Goumans M-J, Doevendans PA. Reduced CD26 expression is associated with improved cardiac function after acute myocardial infarction. Journal of Molecular and Cellular Cardiology. 2012;53:899–905. doi: 10.1016/j.yjmcc.2012.08.026 . 22982114 10.1016/j.yjmcc.2012.08.026

[34] Guilhem A, Malcus C, Clarivet B, Plauchu H. Immunological abnormalities associated with hereditary haemorrhagic telangiectasia. 2013:351–62. doi: 10.1111/joim.12098 23772771 10.1111/joim.12098

[35] Sanz-Rodriguez F, Fernandez-L A, Zarrabeitia R, Perez-Molino A, Ramírez JR, Coto E, et al Mutation analysis in Spanish patients with hereditary hemorrhagic telangiectasia: deficient endoglin up-regulation in activated monocytes. Clinical chemistry. 2004;50:2003–11. doi: 10.1373/clinchem.2004.035287 . 15375013 10.1373/clinchem.2004.035287

[36] Sindrilaru A, Peters T, Wieschalka S, Baican C, Baican A, Peter H, et al An unrestrained proinflammatory M1 macrophage population induced by iron impairs wound healing in humans and mice. Journal of Clinical Investigation. 2011;121:985–97. doi: 10.1172/JCI44490 21317534 10.1172/JCI44490 PMC3049372

[37] Aristorena M, Blanco FJ, de Las Casas-Engel M, Ojeda-Fernandez L, Gallardo-Vara E, Corbi A, et al Expression of endoglin isoforms in the myeloid lineage and their role during aging and macrophage polarization. Journal of cell science. 2014;127:2723–35. doi: 10.1242/jcs.143644 . 24777481 10.1242/jcs.143644

[38] Ojeda-Fernández L, Recio-Poveda L, Aristorena M, Lastres P, Blanco FJ, Sanz-Rodríguez F, et al Mice Lacking Endoglin in Macrophages Show an Impaired Immune Response. PLOS Genetics. 2016;12:e1005935 doi: 10.1371/journal.pgen.1005935 27010826 10.1371/journal.pgen.1005935 PMC4806930

[39] Docherty NG, López-Novoa JM, Arevalo M, Düwel A, Rodriguez-Peña A, Pérez-Barriocanal F, et al Endoglin regulates renal ischaemia-reperfusion injury. Nephrology, dialysis, transplantation: official publication of the European Dialysis and Transplant Association—European Renal Association. 2006;21:2106–19. doi: 10.1093/ndt/gfl179 . 16751653 10.1093/ndt/gfl179

[40] Ervinna N, Mita T, Yasunari E, Azuma K, Tanaka R, Fujimura S, et al Anagliptin, a DPP-4 Inhibitor, Suppresses Proliferation of Vascular Smooth Muscles and Monocyte Inflammatory Reaction and Attenuates Atherosclerosis in Male apo E-Deficient Mice. Endocrinology. 2013;154:1260–70. doi: 10.1210/en.2012-1855 . 23337530 10.1210/en.2012-1855

[41] Röhrborn D, Wronkowitz N, Eckel J. DPP4 in diabetes. Frontiers in Immunology. 2015;6:1–20. doi: 10.3389/fimmu.2015.00386 . 26284071 10.3389/fimmu.2015.00386 PMC4515598

[42] Shinjo T, Nakatsu Y, Iwashita M, Sano T, Sakoda H, Ishihara H, et al DPP-IV inhibitor anagliptin exerts anti-inflammatory effects on macrophages, adipocytes, and mouse livers by suppressing NF-kappaB activation. Am J Physiol Endocrinol Metab. 2015;309(3):E214–23. doi: 10.1152/ajpendo.00553.2014 . 26015438 10.1152/ajpendo.00553.2014

[43] Shah Z, Kampfrath T, Deiuliis Ja, Zhong J, Pineda C, Ying Z, et al Long-term dipeptidyl-peptidase 4 inhibition reduces atherosclerosis and inflammation via effects on monocyte recruitment and chemotaxis. Circulation. 2011;124:2338–49. doi: 10.1161/CIRCULATIONAHA.111.041418 . 22007077 10.1161/CIRCULATIONAHA.111.041418 PMC4224594

[44] Tricco AC, Antony J, Khan PA, Ghassemi M, Hamid JS, Ashoor H, et al Safety and effectiveness of dipeptidyl peptidase-4 inhibitors versus intermediate-acting insulin or placebo for patients with type 2 diabetes failing two oral antihyperglycaemic agents: a systematic review and network meta-analysis. BMJ Open. 2014;4:e005752 doi: 10.1136/bmjopen-2014-005752 . 25537781 10.1136/bmjopen-2014-005752 PMC4275675

[45] Brenner C, Kränkel N, Kühlenthal S, Israel L, Remm F, Fischer C, et al Short-term inhibition of DPP-4 enhances endothelial regeneration after acute arterial injury via enhanced recruitment of circulating progenitor cells. International journal of cardiology. 2014;177:266–75. doi: 10.1016/j.ijcard.2014.09.016 . 25499391 10.1016/j.ijcard.2014.09.016

[46] Zaruba M-M, Zhu W, Soonpaa MH, Reuter S, Franz W-M, Field LJ. Granulocyte colony-stimulating factor treatment plus dipeptidylpeptidase-IV inhibition augments myocardial regeneration in mice expressing cyclin D2 in adult cardiomyocytes. European Heart Journal. 2012;33:129–37. doi: 10.1093/eurheartj/ehr302 21849352 10.1093/eurheartj/ehr302 PMC3249220

[47] Theiss HD, Vallaster M, Rischpler C, Krieg L, Zaruba M-M, Brunner S, et al Dual stem cell therapy after myocardial infarction acts specifically by enhanced homing via the SDF-1/CXCR4 axis. Stem Cell Research. 2011;7:244–55. doi: 10.1016/j.scr.2011.05.003 21752744 10.1016/j.scr.2011.05.003

[48] Rossi E, Smadja DM, Boscolo E, Langa C, Arevalo MA, Pericacho M, et al Endoglin regulates mural cell adhesion in the circulatory system. Cellular and Molecular Life Sciences. 2016;73:1715–39. doi: 10.1007/s00018-015-2099-4 . 26646071 10.1007/s00018-015-2099-4 PMC4805714

[49] Lebrin F, Srun S, Raymond K, Martin S, van den Brink S, Freitas C, et al Thalidomide stimulates vessel maturation and reduces epistaxis in individuals with hereditary hemorrhagic telangiectasia. Nature Medicine. 2010;16:420–8. doi: 10.1038/nm.2131 20364125 10.1038/nm.2131

[50] Mancini ML, Terzic A, Conley BA, Oxburgh LH, Nicola T, Vary CPH. Endoglin plays distinct roles in vascular smooth muscle cell recruitment and regulation of arteriovenous identity during angiogenesis. Developmental Dynamics. 2009;238:2479–93. doi: 10.1002/dvdy.22066 . 19705428 10.1002/dvdy.22066 PMC2947792

[51] Choi SH, Park S, Oh CJ, Leem J, Park KG, Lee IK. Dipeptidyl peptidase-4 inhibition by gemigliptin prevents abnormal vascular remodeling via NF-E2-related factor 2 activation. Vascular Pharmacology. 2015;73:11–9. doi: 10.1016/j.vph.2015.07.005 . 26187356 10.1016/j.vph.2015.07.005

[52] Sauve M, Ban K, Momen MA, Zhou Y-q, Henkelman RM, Husain M, et al Genetic Deletion or Pharmacological Inhibition of Dipeptidyl Peptidase-4 Improves Cardiovascular Outcomes After Myocardial Infarction in Mice. 2010;59 doi: 10.2337/db09-0955.M.H 10.2337/db09-0955 PMC2844815 20097729

[53] Zaruba M-M, Theiss HD, Vallaster M, Mehl U, Brunner S, David R, et al Synergy between CD26/DPP-IV Inhibition and G-CSF Improves Cardiac Function after Acute Myocardial Infarction. Cell Stem Cell. 2009;4:313–23. doi: 10.1016/j.stem.2009.02.013 19341621 10.1016/j.stem.2009.02.013

[54] Brenner C, Adrion C, Grabmaier U, Theisen D, von Ziegler F, Leber A, et al Sitagliptin plus granulocyte colony-stimulating factor in patients suffering from acute myocardial infarction: A double-blind, randomized placebo-controlled trial of efficacy and safety (SITAGRAMI trial). International Journal of Cardiology. 2016;205:23–30. doi: 10.1016/j.ijcard.2015.11.180 26709136 10.1016/j.ijcard.2015.11.180

[55] Gross L, Theiss HD, Grabmaier U, Adrion C, Mansmann U, Sohn H-y, et al Combined therapy with sitagliptin plus granulocyte-colony stimulating factor in patients with acute myocardial infarction—Long-term results of the SITAGRAMI trial. International Journal of Cardiology. 2016;215:441–5. doi: 10.1016/j.ijcard.2016.04.134 27131268 10.1016/j.ijcard.2016.04.134

[56] Savarese G, Perrone-Filardi P, D'Amore C, Vitale C, Trimarco B, Pani L, et al Cardiovascular effects of dipeptidyl peptidase-4 inhibitors in diabetic patients: A meta-analysis. International Journal of Cardiology. 2015;181:239–44. doi: 10.1016/j.ijcard.2014.12.017 25528528 10.1016/j.ijcard.2014.12.017

[57] Zhong J, Maiseyeu A, Davis SN, Rajagopalan S. DPP4 in Cardiometabolic Disease: Recent Insights From the Laboratory and Clinical Trials of DPP4 Inhibition. Circulation Research. 2015;116:1491–504. doi: 10.1161/CIRCRESAHA.116.305665 . 25858071 10.1161/CIRCRESAHA.116.305665 PMC4394189

[58] Frederich R, Alexander JH, Fiedorek FT, Donovan M, Berglind N, Harris S, et al A systematic assessment of cardiovascular outcomes in the saxagliptin drug development program for type 2 diabetes. Postgraduate medicine. 2010;122:16–27. doi: 10.3810/pgm.2010.05.2138 . 20463410 10.3810/pgm.2010.05.2138

[59] Patil HR, Al Badarin FJ, Al Shami HA, Bhatti SK, Lavie CJ, Bell DSH, et al Meta-analysis of effect of dipeptidyl peptidase-4 inhibitors on cardiovascular risk in type 2 diabetes mellitus. The American journal of cardiology. 2012;110:826–33. doi: 10.1016/j.amjcard.2012.04.061 . 22703861 10.1016/j.amjcard.2012.04.061

[60] Cobble ME, Frederich R. Saxagliptin for the treatment of type 2 diabetes mellitus: assessing cardiovascular data. Cardiovascular Diabetology. 2012;11:6 doi: 10.1186/1475-2840-11-6 . 22248301 10.1186/1475-2840-11-6 PMC3277488

[61] Koska J, Sands M, Burciu C, Reaven P. Cardiovascular effects of dipeptidyl peptidase-4 inhibitors in patients with type 2 diabetes. Diab Vasc Dis Res. 2015;12(3):154–63. doi: 10.1177/1479164114562411 . 25852133 10.1177/1479164114562411

[62] Shi S, Srivastava SP, Kanasaki M, He J, Kitada M, Nagai T, et al Interactions of DPP-4 and integrin β1 influences endothelial-to-mesenchymal transition. Kidney International. 2015;88:479–89. doi: 10.1038/ki.2015.103 25830763 10.1038/ki.2015.103

[63] Rinkevich Y, Walmsley GG, Hu MS, Maan ZN, Newman AM, Drukker M, et al Skin fibrosis. Identification and isolation of a dermal lineage with intrinsic fibrogenic potential. Science. 2015;348(6232):aaa2151 doi: 10.1126/science.aaa2151 ; PubMed Central PMCID: PMCPMC5088503. 25883361 10.1126/science.aaa2151 PMC5088503

[64] Thielitz A, Vetter RW, Schultze B, Wrenger S, Simeoni L, Ansorge S, et al Inhibitors of dipeptidyl peptidase IV-like activity mediate antifibrotic effects in normal and keloid-derived skin fibroblasts. Journal of Investigative Dermatology. 2007;128:855–66. doi: 10.1038/sj.jid.5701104 17943180 10.1038/sj.jid.5701104

[65] Grilo GA, Shaver PR, de Castro Bras LE. The Prospective Cardioprotective Effects of DPP-4 inhibition in the ischemic myocardium. J Mol Cell Cardiol. 2016;93:44–6. doi: 10.1016/j.yjmcc.2016.01.021 . 26916446 10.1016/j.yjmcc.2016.01.021

[66] Choi HJ, Kim JY, Lim SC, Kim G, Yun HJ, Choi HS. Dipeptidyl peptidase 4 promotes epithelial cell transformation and breast tumourigenesis via induction of PIN1 gene expression. Br J Pharmacol. 2015;172(21):5096–109. doi: 10.1111/bph.13274 ; PubMed Central PMCID: PMCPMC4687806. 26267432 10.1111/bph.13274 PMC4687806

[67] Zhu X, Gillespie DG, Jackson EK. NPY 1–36 and PYY 1–36 activate cardiac fibroblasts: an effect enhanced by genetic hypertension and inhibition of dipeptidyl peptidase 4. American Journal of Physiology—Heart and Circulatory Physiology. 2015;309:H1528–H42. doi: 10.1152/ajpheart.00070.2015 26371160 10.1152/ajpheart.00070.2015 PMC4666977

[68] Gangadharan Komala M, Gross S, Zaky A, Pollock C, Panchapakesan U. Linagliptin Limits High Glucose Induced Conversion of Latent to Active TGFß through Interaction with CIM6PR and Limits Renal Tubulointerstitial Fibronectin. Plos One. 2015;10:e0141143 doi: 10.1371/journal.pone.0141143 26509887 10.1371/journal.pone.0141143 PMC4624988

[69] Kanasaki K, Shi S, Kanasaki M, He J, Nagai T, Nakamura Y, et al Linagliptin-Mediated DPP-4 Inhibition Ameliorates Kidney Fibrosis in Streptozotocin-Induced Diabetic Mice by Inhibiting Endothelial-to-Mesenchymal Transition in a Therapeutic Regimen. Diabetes. 2014;63:2120–31. doi: 10.2337/db13-1029 24574044 10.2337/db13-1029

[70] Panchapakesan U. DPP4 Inhibition in Human Kidney Proximal Tubular Cells—Renoprotection in Diabetic Nephropathy? Journal of Diabetes & Metabolism. 2013;S9:1–8. doi: 10.4172/2155-6156.S9-007 10.1371/journal.pone.0054442 PMC3563635 23390498

