# Supplementary figures and images for: Inhibiting DPP4 in a mouse model of HHT1 results in a shift towards regenerative macrophages and reduces fibrosis after myocardial infarction
Source: PLoS One. 2017 Dec 18;12(12):e0189805. doi: 10.1371/journal.pone.0189805 (PMC5734765; doi:10.1371/journal.pone.0189805)

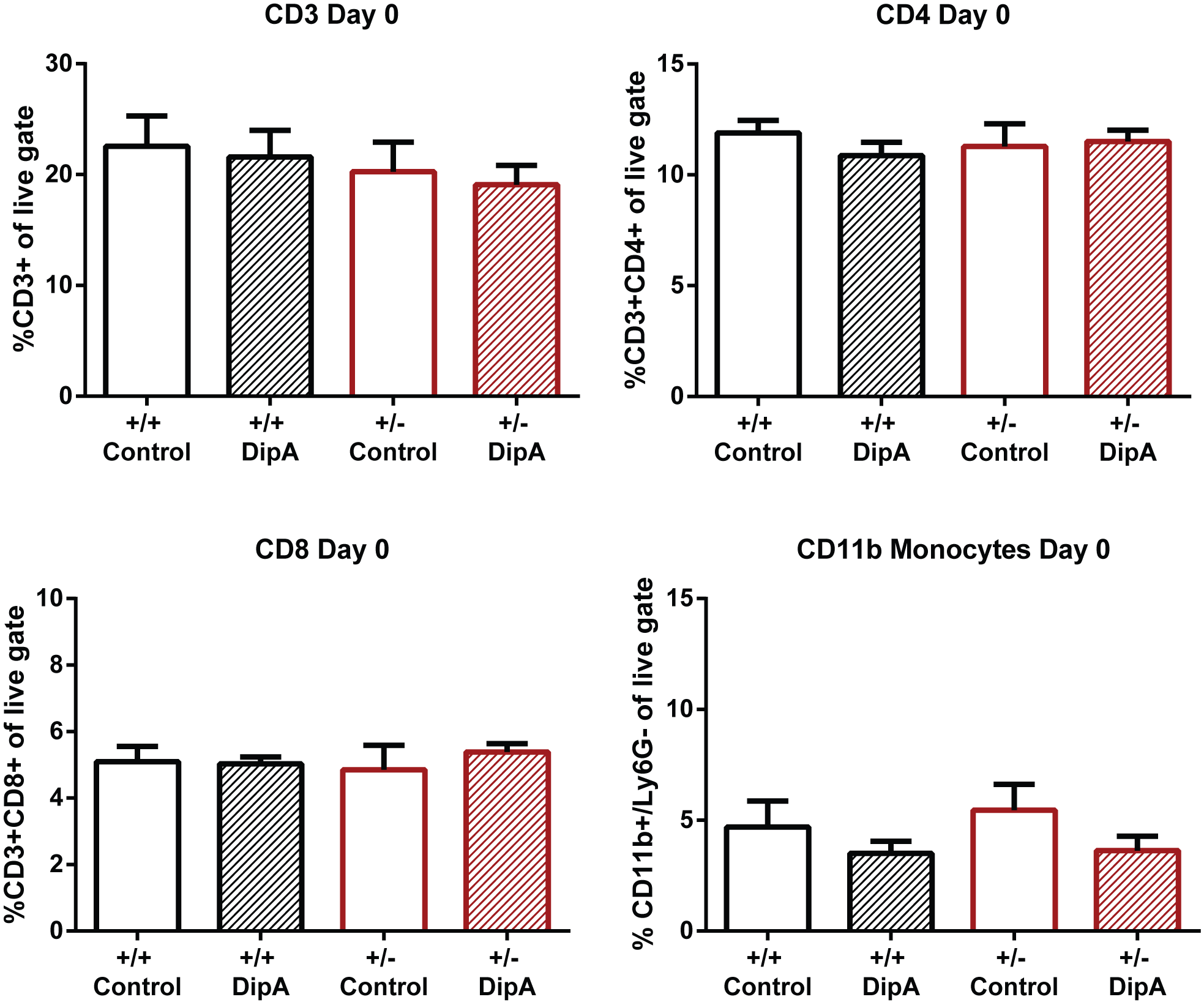

Supplement: S1 Fig — Flow cytometric analysis of the major leukocyte subsets in the circulation of the mice groups at day 0 (pre-MI and pre-DipA treatment). Leukocytes labeled for anti-mouse CD3, CD3+/CD4+, CD3+/CD8+ and CD11b+/Ly6G- (n = 3–6, non-parametric ANOVA testing). Control = MQ treated, DipA = Diprotin A treated group. Data shown are mean ± SEM, *P<0.05. (TIF) [file pone.0189805.s001.tif]

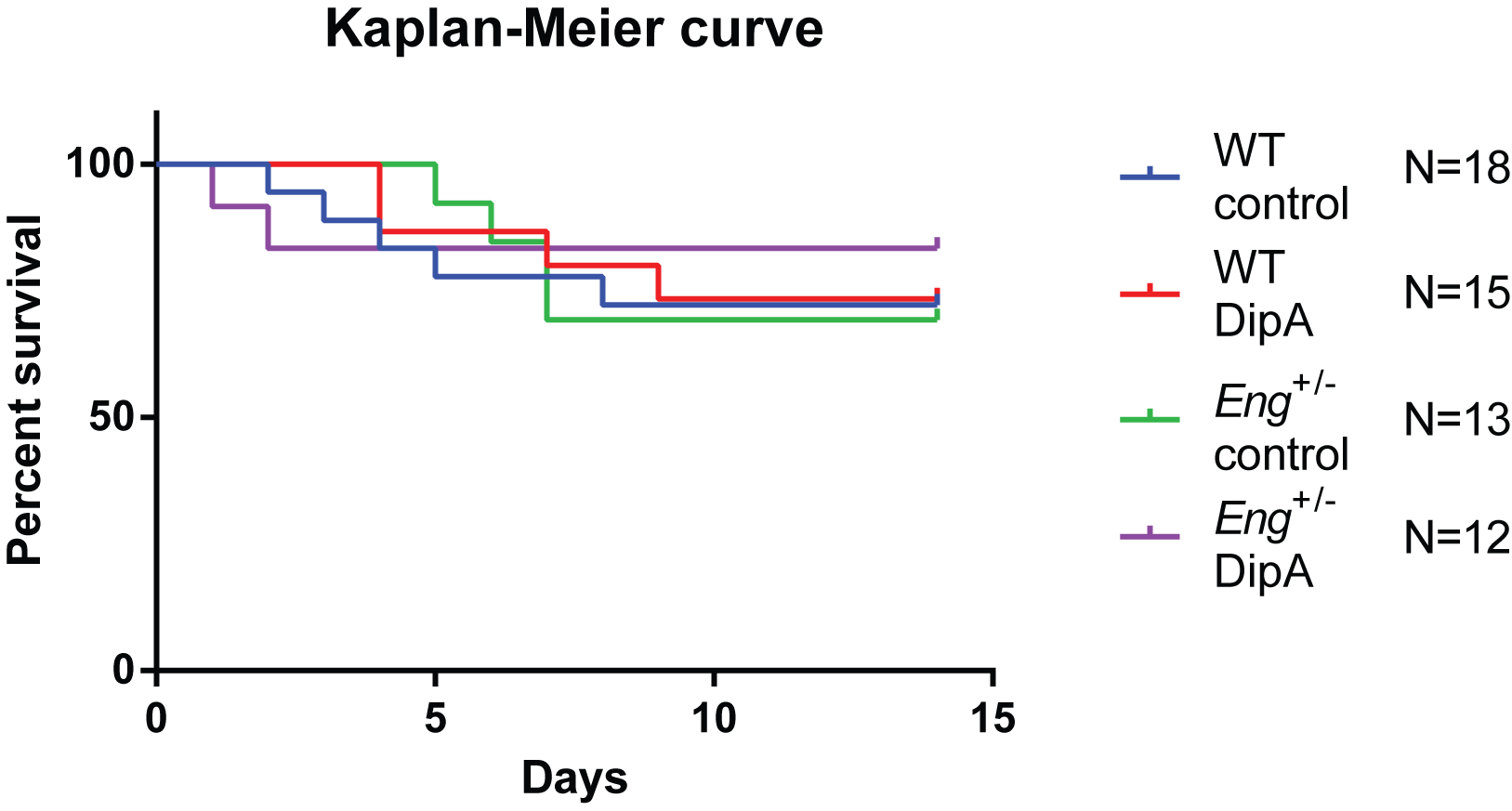

Supplement: S2 Fig — Kaplan-Meier curve of WT and Eng+/- mice 14 days post-MI. Graph depicts percentage of surviving WT and Eng+/- mice, control and DipA treated animals (n = 12–18). Control = MQ treated, DipA = Diprotin A treated group. (TIF) [file pone.0189805.s002.tif]

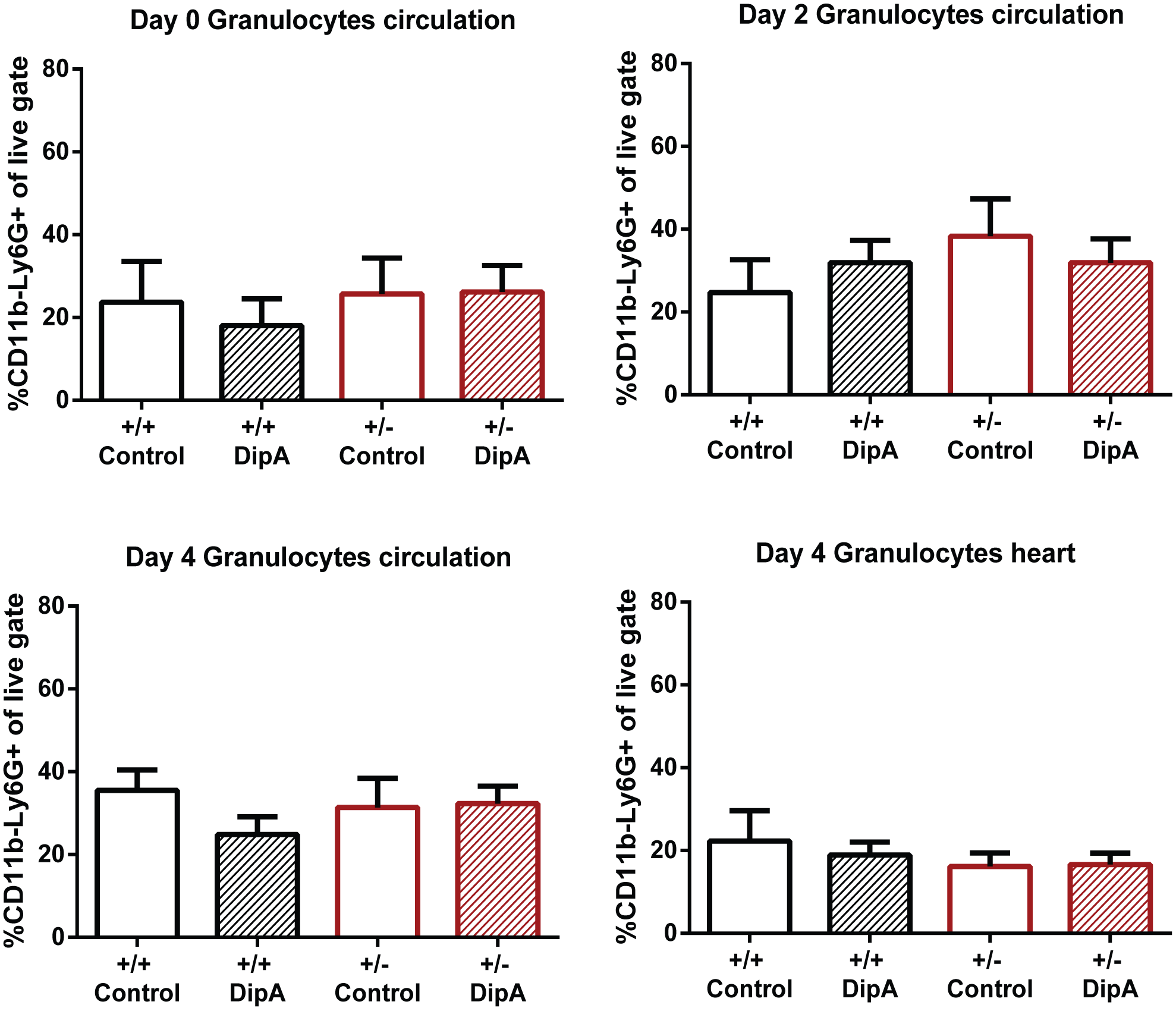

Supplement: S3 Fig — Flow cytometric analysis of the granulocyte subset in the circulation of the mice at (A) day 0 (pre-MI and pre-DipA treatment) and (B) day 2 and (C) 4 post-MI in the circulation. (D) Granulocytes isolated from the infarcted part of the LV 4 days post-MI. Leukocytes labeled with anti-mouse CD11b, Ly6G. Granulocytes were identified as the CD11b-/Ly6G+ population of the live gate (n = 3–6, non-parametric ANOVA testing). Control = MQ treated, DipA = Diprotin A treated group. Data shown are mean ± SEM, *P<0.05. (TIF) [file pone.0189805.s003.tif]

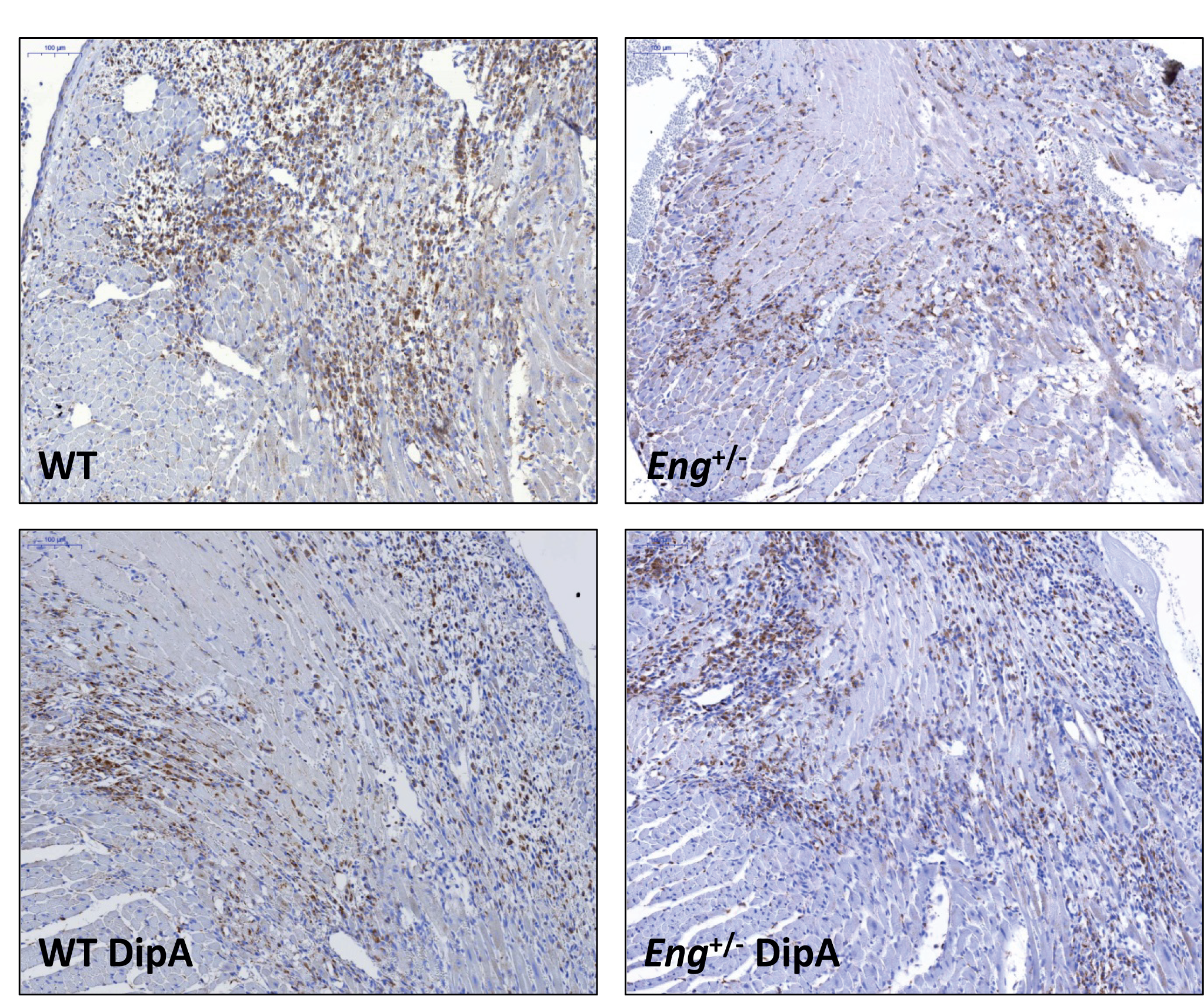

Supplement: S4 Fig — Transversal sections of mouse hearts were stained for macrophage marker MAC3 using immunohistochemistry (n = 6–7). Photos taken at 15x magnification. MAC3 = brown, nuclei = blue. (TIF) [file pone.0189805.s004.tif]

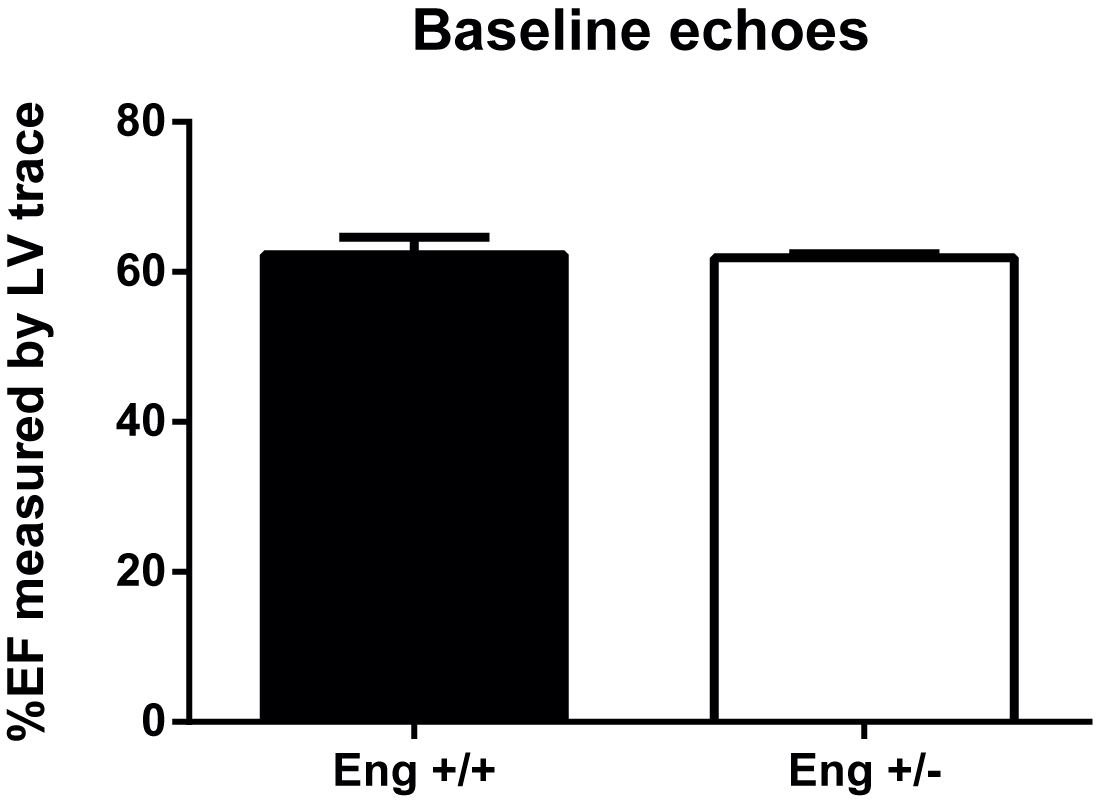

Supplement: S5 Fig — Baseline cardiac function in percent ejection fraction (%EF) between WT and Eng+/- mice (n = 4). Cardiac function was measured by ultrasound. Data shown are mean ± SEM, *P<0.05. (TIF) [file pone.0189805.s005.tif]

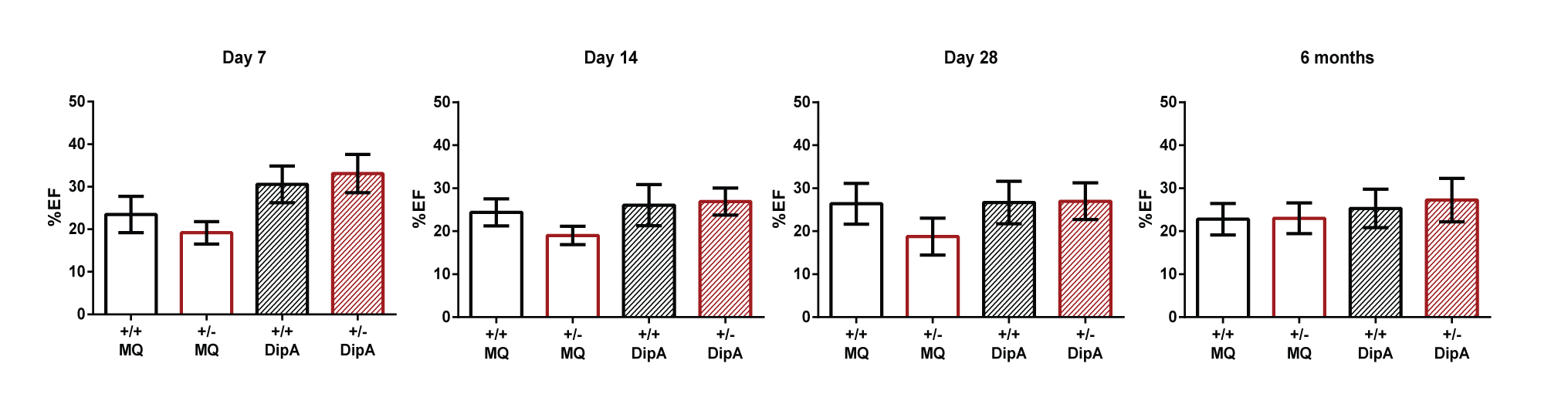

Supplement: S6 Fig — Percentage EF 7, 14, 28 days and 6 months post-MI of WT(+/+) and Eng+/- (+/-) mice, control (Milli-Q ultrapure sterile water, MQ) and DipA treated animals. EF was measured by ultrasound and analyzed by left ventricle tracing (n = 5–11). Data shown are mean ± SEM, *P<0.05. (TIF) [file pone.0189805.s006.tif]

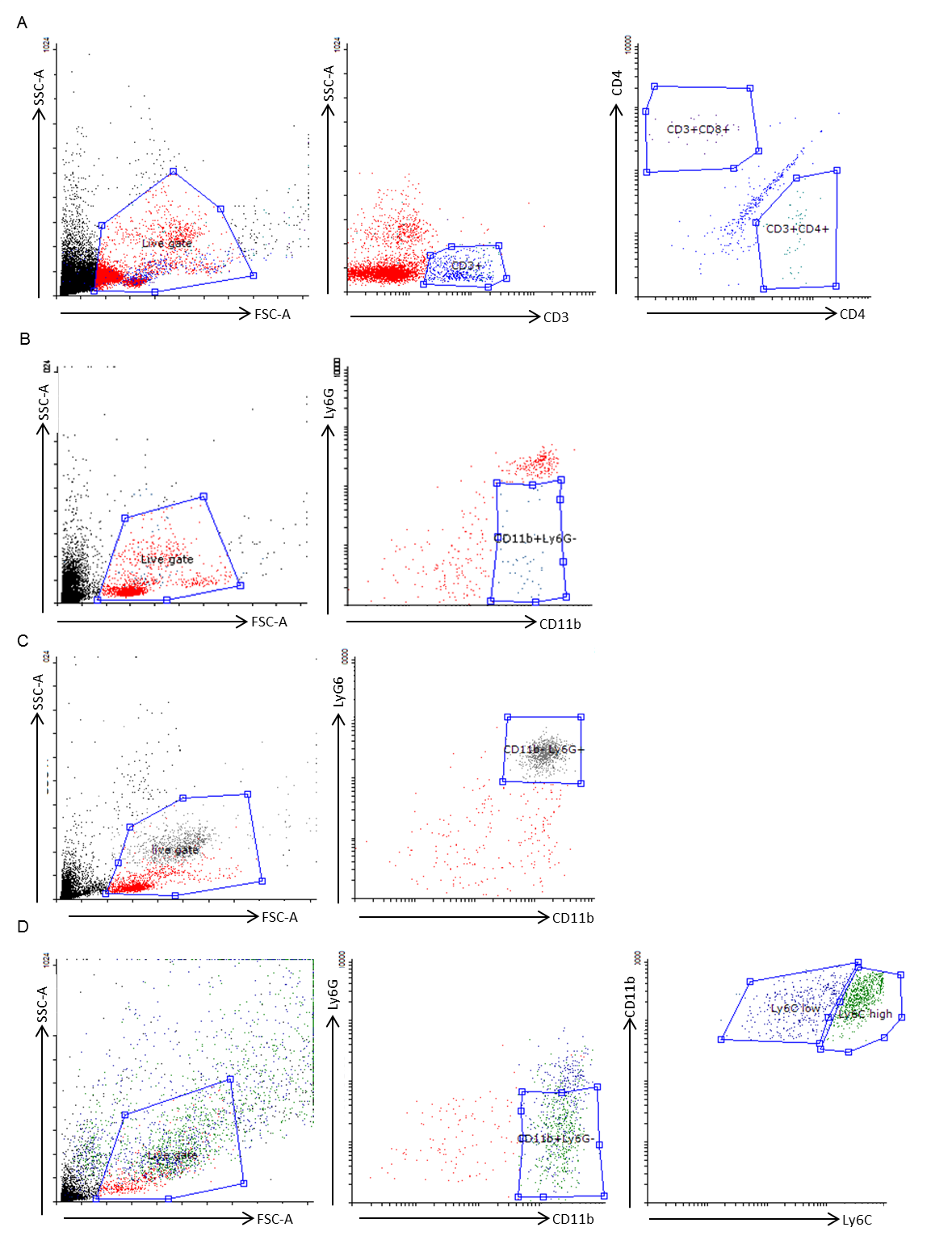

Supplement: S7 Fig — (A) Gating strategy for CD3, 4 and 8 T-cells in the blood. The first gating step is a gate for live cells using FSC and SSC. T-cells are subsequently identified using CD3. T-cells subsets are then identified with CD4 and CD8. (B) Monocytes gating strategy in the blood. The first gating step is a gate for live cells using FSC and SSC. The second gating step is for monocytes, identified by CD11b positive and Ly6G negative labeling. (C) Granulocyte gating strategy in the blood. The first gating step is a gate for live cells using FSC and SSC, the granulocytes are subsequently identified by Ly6G. (D) Macrophage subsets gating strategy from MNCs isolated from the left ventricle. The first gating step is a gate for live cells using FSC and SSC, the monocytes are identified by CD11b positive and Ly6G negative labeling. The inflammatory-like M1 macrophages are then subsequently identified by Ly6Chigh and regenerative-like M2 macrophages identified by Ly6Clow labeling. (TIF) [file pone.0189805.s007.tif]
